# Everything You Always Wanted to Know About *Salmonella* Type 1 Fimbriae, but Were Afraid to Ask

**DOI:** 10.3389/fmicb.2019.01017

**Published:** 2019-05-14

**Authors:** Rafal Kolenda, Maciej Ugorski, Krzysztof Grzymajlo

**Affiliations:** Department of Biochemistry and Molecular Biology, Faculty of Veterinary Medicine, Wrocław University of Environmental and Life Sciences, Wrocław, Poland

**Keywords:** *Salmonella*, type 1 fimbriae, adhesion, invasion, regulation of expression

## Abstract

Initial attachment to host intestinal mucosa after oral infection is one of the most important stages during bacterial pathogenesis. Adhesive structures, widely present on the bacterial surface, are mainly responsible for the first contact with host cells and of host-pathogen interactions. Among dozens of different bacterial adhesins, type 1 fimbriae (T1F) are one of the most common adhesive organelles in the members of the *Enterobacteriaceae* family, including *Salmonella* spp., and are important virulence factors. Those long, thin structures, composed mainly of FimA proteins, are responsible for recognizing and binding high-mannose oligosaccharides, which are carried by various glycoproteins and expressed at the host cell surface, via FimH adhesin, which is presented at the top of T1F. In this review, we discuss investigations into the functions of T1F, from the earliest work published in 1958 to operon organization, organelle structure, T1F biogenesis, and the various functions of T1F in *Salmonella*-host interactions. We give special attention to regulation of T1F expression and their role in binding of *Salmonella* to cells, cell lines, organ explants, and other surfaces with emphasis on biofilm formation and discuss T1F role as virulence factors based on work using animal models. We also discuss the importance of allelic variation in *fimH* to *Salmonella* pathogenesis, as well as role of FimH in *Salmonella* host specificity.

## Introduction

**FIGURE 1:**
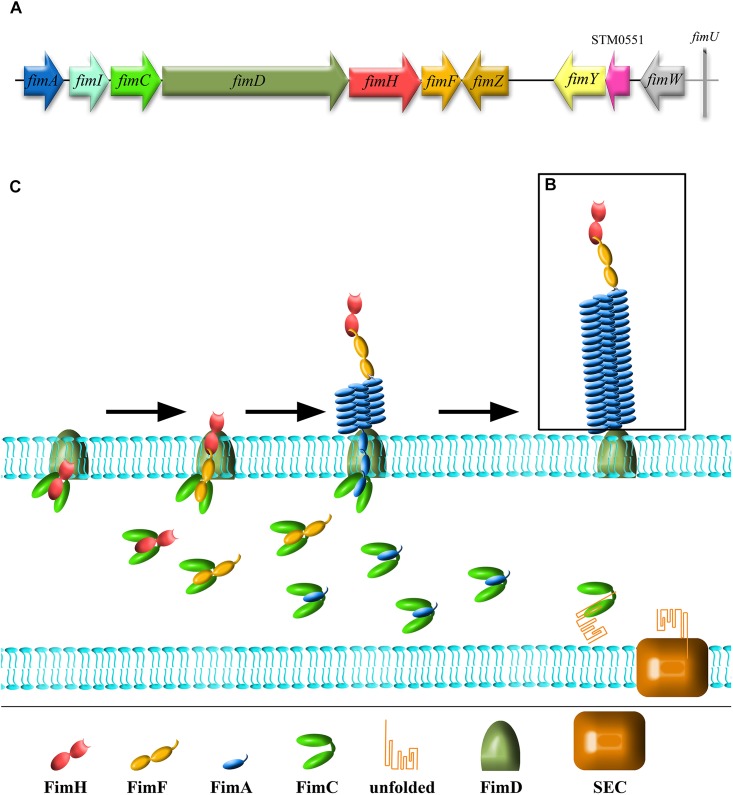
Schematic representation of *Salmonella* type 1 fimbriae (T1F). **(A)** *fim* gene cluster organization, **(B)** structure of fimbrium, and **(C)** biogenesis by the chaperon-usher pathway.

Adhesion to host tissues is thought to be one of the crucial events during *Salmonella* pathogenesis. Among dozens of different bacterial adhesins, type 1 fimbriae (T1F) are one of the most common adhesive organelles in the members of the *Enterobacteriaceae* family, including *Salmonella* spp., and are important virulence factors. The *fim* fimbrial cluster is one of seven most abundant fimbrial clusters (including *fim, bcf*, *stb*, *sth*, *std*, *saf*, and *sti*) in the genome of *Salmonella* spp. (Yue et al., 2012). The importance of T1F in *Salmonella* biology is confirmed by the fact that it is expressed in more than 80% of 1453 clinical isolates, representing 149 serovars (Duguid et al., 1966). The *Salmonella* *fim* cluster comprises 10 genes (*fimA, fimI, fimC, fimD, fimH, fimF, fimZ, fimY, fimW*, and *stm0551*) and an tRNA-Arg (Purcell et al., 1987; Boyd and Hartl, 1999). Of these, *fimA*, *fimI*, *fimC*, *fimD*, *fimH*, and *fimF*, comprise a single operon, under the control of *fimA* promoter region (P_fimA_). The six genes in this operon encode proteins involved in biogenesis and structure of T1F (Figure 1A). FimW, FimY, FimZ proteins and the STM0551 open reading frame are involved in transcriptional regulation of T1F and tRNA-Arg additionally controls expression of T1F on translational level. T1F are rod shaped structures composed of primarily 500 to 3000 FimA monomers (Hahn et al., 2002), with a single lectin-like protein, FimH, which is directly involved in the binding of high-mannose oligosaccharides carried by surface glycoproteins of eukaryotic cells and is placed on the top of the fimbrial shaft by FimF (Figure 1B).

Type 1 fimbriae are assembled by the chaperone-usher pathway (for a detailed review see Waksman and Hultgren, 2009; Werneburg and Thanassi, 2018; Figure 1C). All proteins needed for the assembly of T1F contain signal peptides. FimC acts as a chaperone for FimA, FimF and FimH, preventing premature polymerization in the periplasm, and takes part in folding and assembly of the fimbriae. FimA, FimF, and FimH contain hydrophobic N- and C-terminal extensions that are bound by a complementary hydrophobic groove in FimC. FimD is an usher outer-membrane protein that exports fimbrial proteins through the outer membrane and facilitates fimbriae subunit assembly. All of the proteins that constitute T1F are linked together by N- and C-terminal extensions, a process referred to as donor strand exchange (Remaut et al., 2006). T1F assembly starts with the binding of the FimC-FimH complex to the FimD usher protein. The FimC–FimF complex is next transferred into the FimD pocket, and the FimC bound to the C-terminal extension of FimH is exchanged for the N-terminal extension of FimF, resulting in the formation of the FimH–FimF complex. In the next step, donor strand exchange is repeated with FimA and further elongation of the fimbrial shaft is continued with FimA. Deletion of any one of *fimA*, *fimF* or *fimH* results in no fimbriae production (Zeiner et al., 2012), ascribing a shared role for all of these genes in pilus biogenesis. Though it has been speculated that *fimI* is required for regulation of fimbriae length, and therefore adhesion, the mechanism of this process remains unknown (Rossolini et al., 1993).

Since the first publication in 1958 describing T1F in *Salmonella*, 150 studies concerning this topic have been published consistently, showing constant interest of scientific community in this virulence factor (Supplementary Figure 1). In this review, we summarize current knowledge on the regulation of *Salmonella* T1F expression, the roles of different T1F encoding genes in virulence, and discuss perspectives of future work in this field.

## Early Studies on T1F

The occurrence of T1F in *Salmonella* spp. was first described by Duguid and Gillies (Duguid and Gillies, 1958). This initial study focused mainly on the ability of different *Salmonella* serovars and isolates to produce fimbriae and on conditions that induced or inhibited T1F expression. Moreover, the authors analyzed agglutination of red blood cells (RBCs) isolated from different animal species caused by T1F-positive (T1F+) *Salmonella*, and indicated, for the first time, that *Salmonella* Gallinarum produces T1F that do not agglutinate RBCs from all species tested in this study (Supplementary Table 1). Follow-up experiments conducted by Duguid provided information on the fimbrial status of 149 serovars and 1442 isolates, and showed for the first time the mannose-dependent agglutination of RBCs (Duguid et al., 1966). Both studies revealed that induction of T1F in static liquid culture led to pellicle formation, and multiple passages of bacteria in these conditions usually led to an increase in the fraction of T1F+ bacteria. On the other hand, growth on solid agar resulted in nearly no T1F+ *Salmonella*. In another study from Duguid’s lab, it was observed that T1F mediates adhesion of *Salmonella* to RBCs, leukocytes and epithelial cells. That study also found that induction of T1F expression in *Salmonella* was associated with growth in static aerobic conditions for 24–48 h, with multiple passages leading to an increase in the fraction of T1F+ bacteria (Old and Duguid, 1970).

## Regulation of T1F Expression

The aforementioned work revealed the impact of *Salmonella* growth conditions on T1F expression. It was later shown that, depending on environmental conditions, T1F expression undergoes phase variation (Silverman et al., 1979), and is either in the “off” phase, wherein the whole operon is not transcribed, or in the “on” phase, which results in the expression of *fim* operon mRNA. Expression state is heritable but also reversible, with the frequency of switching from “on” to “off” much higher (approx. 10^-2^ per generation) than the switch in the reverse direction (approx. 10^-4^ per generation; Isaacson and Kinsel, 1992; Isaacson et al., 1999; Patterson et al., 2012). The ability to switch fimbriae expression from the “on” to “off” state and vice-versa plays an important role in *Salmonella* pathogenesis, since presence or absence of these structures on the surface of bacteria can affect various stages of bacterial infection (see below). The mechanisms of phase variation among different bacterial species are diverse, and in the family *Enterobacteriaceae*, such mechanisms are clearly described only in *E. coli*. Moreover, despite morphological and functional similarities of T1F in *E. coli* and *Salmonella*, they are serologically (Clegg et al., 1985) and evolutionarily (Kisiela et al., 2013) unrelated. It is therefore not surprising that the regulatory mechanisms of *fim* operon phase variation in *Salmonella* are quite different from those in the *E. coli*. Based on phase variation experiments with *Salmonella* Typhimurium strain 798, Patterson et al. (2012) hypothesized, that phase variation controls not only expression of T1F but also regulates expression of determinants responsible for invasion, intracellular survival, O-antigen chain length, and sensitivity to complement.

### Direct Regulation

**FIGURE 2:**
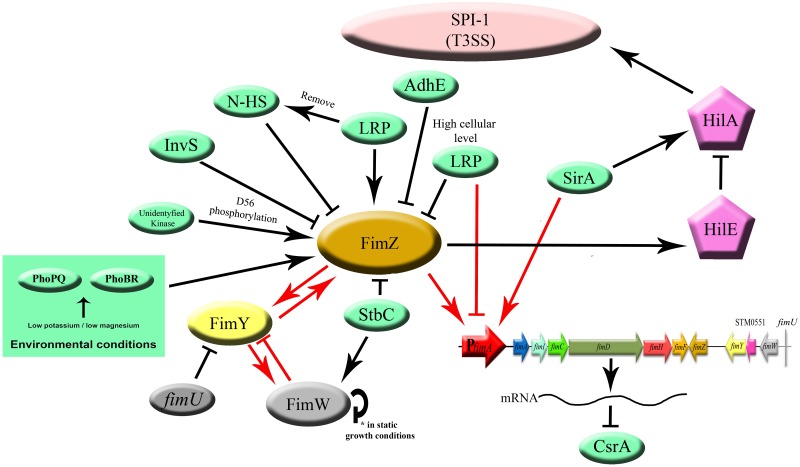
Regulation of T1F expression in *Salmonella*. Proteins directly involved in T1F expression are represented as ovals. Proteins involved in activation of T3SS (HilE, HilA) are represented as pentagons. All proteins encoded by the *fim* operon, including three major regulatory proteins, FimZ, FimY, and FimW are colored according to the scheme in the operon figure (Figure 1). Red arrows indicate direct promoter activation. Black arrows indicate another type of activation (not specified).

In *Salmonella*, there are three major regulatory proteins, FimZ, FimY, and FimW (each expressed under its own promoter), that control *fim* operon expression primarily through regulation of the *fimA* promotor (P*_fimA_*; Yeh et al., 1995, 2002b; Tinker and Clegg, 2000, 2001). FimZ and FimY are both necessary for positive regulation of T1F expression (Yeh et al., 1995; Tinker and Clegg, 2000), with FimZ as a dominant activator (Saini et al., 2009), whereas FimW was found to be a negative regulator (Tinker et al., 2001; Figure 2). Additionally, a putative phosphodiesterase encoded by an open reading frame, *stm0551*, that is located between *fimY* and *fimW*, was found to down-regulate T1F expression in *S.* Typhimurium (Wang et al., 2012). At the end of *fim* cluster, a tRNA^Arg^ encoded by *fimU* is present.

FimZ, which is thought to be a dominant activator of T1F expression, has high homology to a family of DNA binding proteins associated with response regulators of two-component regulatory systems and is able to bind the region upstream (from –47 to –98 nucleotides) of the *fimA* transcription initiation site, similar to other classical activators (Yeh et al., 2002b). It has been proposed that initial activation of FimZ could be driven by phosphorylation of aspartate 56 (D56; Zeiner et al., 2013) mediated by a yet unidentified kinase (Saini et al., 2009). Constitutive phosphorylation stemming from D56E mutation resulted in fimbriated bacteria even under non-inducing conditions. On the other hand, a null phenotype (D56A) blocked fimbriation completely. The authors suggested 31 putative sensory kinases that can phosphorylate FimZ at D56, however, this phosphorylation has not been directly linked to any one of these kinases. FimZ is able to regulate its own expression (Yeh et al., 2002a; Saini et al., 2009), however, no binding of FimZ to its own regulatory region was detected, and therefore the mechanism of such regulation is still uncertain. Moreover, FimZ is considered to be an important control protein of other *Salmonella* regulatory systems and coordinates different aspects of infection.

Activation of the *fimA* promoter by FimZ requires the presence of a second regulator – FimY (Yeh et al., 1995), which acts upstream of FimZ in the regulatory pathway, but does not interact directly with either FimZ or FimW (Zeiner et al., 2013). FimY and FimZ were found to cross activate each other’s promotor regions (P*_fimY_* and P*_fimZ_*), and then, through accumulation of both proteins, strongly stimulate T1F expression (Saini et al., 2009). Interestingly, *fimZ* cloned into a multicopy plasmid can overcome the lack of FimY (Zeiner, 2012). On the other hand, FimY is able to induce expression of *fimW*, which results in repression of P*_fimY_*. Wang et al. (2014) provided evidences that the FimY of *S.* Typhimurium is a DNA-binding protein and binds to a region within the *fimZ* promoter (Wang et al., 2014).

A third major regulatory protein, FimW, acts as a repressor of T1F expression and was found to be highly expressed in conditions favoring poor or no *Salmonella* fimbriation (i.e., growth on solid medium). Moreover, FimW was found to auto-regulate its own expression, but the exact mechanism is not known (Tinker et al., 2001). As with FimY, there has been no demonstrated direct interaction between FimW and the *fimA* promoter region, despite the presence of a putative DNA binding site (Clegg et al., 2011). However, direct interactions with FimZ have been observed (Zeiner et al., 2013). The authors suggested that FimW may have a modulatory role in FimZ-induced activation of the T1F *fimA* promoter by preventing FimZ binding to P*_fimA_*. According to Saini et al. (2009), expression of two T1F activators, FimZ and FimY, is negatively controlled by FimW, since FimY activates P*_fimW_* and, therefore, initiates a negative feedback loop.

In 1986, Feutrier et al. (1986) reported that T1F expression can be regulated by a Tn*10* element, later recognized as the *fimU* coding tRNA^Arg^, which has an impact on translation of *fim* regulatory genes (Clouthier et al., 1998). This tRNA is indispensable for efficient translation of *fimY* mRNA, which contains a number of rare AGA and AGG arginine codons that are recognized by the tRNA^Arg^ (Swenson et al., 1994; Clouthier et al., 1998). There are five rare arginine AGA codons in T1F – three in *fimY* and two in *fimW* – however, *fimU* deficiency only affects expression of FimY (Tinker and Clegg, 2001).

### Global Regulation

*Salmonella* T1F expression can be also regulated by transcription factors involved in global regulation of genes engaged in metabolism, stress response or production of virulence factors. So far, the protein products of *lrp*, *sirA, iprA, stbC, yqiC*, *invS*, *arcZ*/*hfq*, and *adhE* have been implicated in T1F regulation (Figure 2).

Leucine responsive regulatory protein (Lrp) is an 18.8 kDa DNA binding protein known to regulate many fimbrial genes and to induce the *E. coli fim* operon (Blomfield et al., 1993). McFarland et al. (2008) reported that Lrp-negative mutants of *S.* Typhimurium do not express T1F. Lrp directly affects *fimZ* expression, probably by displacement of histone-like nucleoid-structuring (N-HS) protein, a global repressor of gram-negative bacteria (reviewed in Dorman, 2004), which binds to *fimZ* promoter via AT-rich sequences (Navarre et al., 2006). More recent studies revealed that direct binding of Lrp to the *fimA* promoter is indispensable for activation as well as repression of T1F expression (Baek et al., 2011). This activation or repression depends on cellular Lrp levels, as excessive or insufficient levels of Lrp results in inhibition of T1F expression. The same study also showed that Lrp expression is directly related to the nutritional conditions of the environment, and that T1F expression can be linked to cellular nutritional status via Lrp levels. *sirA* involved in *Salmonella* biofilm formation also takes part in positive regulation of T1F expression (Teplitski et al., 2006). Since SirA can directly bind to P*_fimA_*, it was hypothesized that this protein is necessary for initiation of *fim* operon transcription and increases the expression of T1F. *iprA* in *S.* Typhimurium, another gene that increases the expression of T1F, is highly conserved among *Enterobacteriaceae*. A deletion mutant of *iprA* showed decreased expression of the *fim* operon and fimbrial *stbB, stcB, stdA*, and *stfE*, and increased expression of *stbA* (Herman et al., 2016).

All abovementioned genes positively regulate T1F expression, but several other genes expressed by *Salmonella* suppress T1F expression. It was shown that deletion of *yqiC* (Wang et al., 2016) or *arcZ* (Monteiro et al., 2012) activates T1F expression in *S.* Typhimurium . *arcZ* and *stbC* are examples of cross-talk between different fimbrial systems (Monteiro et al., 2012; Wu et al., 2012). ArcZ, a protein involved in biofilm formation, plays a role in T1F expression by mediating the switch between T1F and curli fimbriae expression, as upregulation of T1F expression was observed in an *arcZ* deletion mutant (Papenfort et al., 2009; Monteiro et al., 2012). The *stbC* gene product, an Stb fimbriae usher, also plays a role in the T1F regulatory network. An *stbC* mutant had higher *fimZ* expression and lower *fimW* expression, and therefore stimulated T1F production. Depending on environmental conditions, T1F expression can also be affected by other factors, e.g., by proteins like AdhE (fermentative alcohol dehydrogenase) that are involved in carbohydrate metabolism (Chuang et al., 2008). Overexpression of AdhE is known to inhibit T1F expression, whereas the absence of AdhE stimulates T1F expression even in *Salmonella* growing on solid agar.

### Crosstalk Between T1F and Other Virulence Factors

Expression of T1F can have an impact on expression of other virulence factors. It was shown that the activated *fim* operon prevents expression of plasmid-encoded fimbriae (Pef) by regulating CsrA activity, demonstrating a hierarchy in expression of different types of fimbriae (Sterzenbach et al., 2013). Binding of the polycistronic *fimAICDHF* mRNA to CsrA, a global post-transcriptional regulator, decreases CsrA activity, and thereby inhibits its positive effect on Pef expression (Figure 2).

FimZ, involved in direct regulation of *fim* operon, is also involved in regulation of motility. FimZ overexpression correlates with decreased *Salmonella* motility and invasion (Clegg and Hughes, 2002), as the *Salmonella* invasion process requires precisely regulated, hierarchical expression of different proteins. The invasion phenotype is dependent on the activation of *Salmonella* Pathogenicity Island 1 Type 3 Secretion System (SPI-1 T3SS) by a variety of positive and negative regulatory genes, with *hilA* and *invF* as two key activators (Lee et al., 1992; Bajaj et al., 1995). It was shown that *hilA* expression can be regulated by the level of *hilE* expression, and *hilE* is regulated by *fimY* and *fimZ* (Baxter and Jones, 2005). Induction of *hilE*, stimulated by *fimYZ*, leads to repression of *hilA* and subsequent down-regulation of invasion genes encoded by SPI-1 (Figure 2).

Recent work has shown that two-component regulatory systems, such as PhoPQ and PhoBR, are able to activate *fimZ*, and therefore to increase T1F expression, as well as upregulate expression of *hilE*. As activation of PhoPQ and PhoBR is induced by low magnesium and low phosphate concentrations, respectively, it directly links regulation of the *fim* operon with environmental conditions (Baxter and Jones, 2015). PhoPQ, together with several other regulatory systems (SsrA/B, OmpR/EnvZ), positively regulates InvS expression (Colgan et al., 2016), a *Salmonella* small RNA essential for invasion (Wang et al., 2017). InvS reduces expression of *fimZ* and increases expression of *flhD*, an important regulator of flagella expression, but the exact mechanism is still unclear. Saini et al. (2010) proposed that the three systems involved in *Salmonella* infection are subject to dynamic regulation. First, *Salmonella* use flagella to swim to site of invasion, then SPI-1 T3SS is expressed and finally T1F are expressed. The authors suggested that the most significant regulator of these processes is the flagellar regulator FliZ, as it regulates both SPI-1 and T1F expression; however, no direct effect of FliZ on *fim* cluster expression was observed.

Taking together, it seems that specific environmental signals can promote *Salmonella* fimbrial phase variations, and that this process is specifically related to virulence. What is more, a main regulator of T1F expression, FimZ, plays an important role not only in fimbrial phase regulation, but also acts as a one of the global regulons of a wide range of phenotypes in various stages of *Salmonella* infection.

## T1F and Adhesion of *Salmonella* to Cells, Organ Explants, Cell Lines and Their Role in Biofilm Formation

The role of T1F in adhesion of *Salmonella* to eukaryotic cells has been extensively studied. Work on T1F-dependent binding in *Salmonella* was conducted primarily using yeast agglutination and guinea pig hemagglutination as controls for T1F expression (Supplementary Table 1). Other often used models are primary animal cell cultures or organ explants (Supplementary Table 1). For example, *S.* Typhimurium adhesion to and infection of isolated rat small intestine enterocytes occurred in a mannose-sensitive (MS) manner, suggesting the involvement of T1F (Lindquist et al., 1987). Similar MS adhesion by T1F+ *S.* Typhimurium was also reported in isolated intestines of 1-day old chicks as well as isolated rat enterocytes (Oyofo et al., 1989). Adhesion of *Salmonella* Enteritidis to human buccal cells and mouse small intestine epithelial cells was mediated by T1F and blocked by preincubation of bacteria with D-mannose (Aslanzadeh and Paulissen, 1990). However, when the *S.* Enteritidis wild-type (WT) strain and a *fimD* deletion mutant were analyzed for adhesion to chicken duodenal explants, no significant difference between these two strains was found (Allen-Vercoe and Woodward, 1999). Dendritic cells (DC) can send dendrites between enterocytes, which enables direct contact of DC with *Salmonella*. Using T1F+ *S.* Typhimurium, it was found that bacteria can bind to murine bone marrow-derived DCs in a MS manner (Guo et al., 2007).

There are 20 articles on *Salmonella* T1F-dependent binding to established *in vitro* cell lines (Table 1). It should be mentioned that the majority of these studies were performed with human non-intestinal cell lines, such as epithelioid cervix carcinoma Hela cells and HeLa derivative HEp-2 cells. Studies on *S.* Typhimurium and *Salmonella* Braenderup have shown that T1F+ strains adhered to and invaded HeLa cells in higher numbers than non-fimbriated strains (Horiuchi et al., 1992; Bäumler et al., 1996). Many studies with HEp-2 cells have shown that *Salmonella* also adhere to these cells in a MS manner, and therefore such binding is mediated by T1F (Tavendale et al., 1983; Old et al., 1986; Ernst et al., 1990). In a study by Hancox et al. (1997), WT *S.* Typhimurium bound better to the HEp-2 and HeLa cell lines than isogenic *fimH* mutants. However, according to early investigations conducted by Jones and Richardson (1981), *S.* Typhimurium adhesion and invasion to HeLa cells occurred in a mannose-resistant (MR) manner, and therefore was probably not mediated by T1F. Furthermore, results presented by Bäumler et al. (1996) showed that T1F did not contribute to adhesion and invasion of *S.* Typhimurium to HEp-2 cells.

**Table 1:** Summary of assays performed to study the role of T1F in *Salmonella* adhesion and/or invasion of cell lines.

No.	Serovar	Strains	Bacteria- growth conditions	Cell line and growth conditions	Outcome	Source
1.	Typhimurium	TML, W118, NY, PR (T1F+); S850, S2204 (T1F-)	Temp: ? Time: ? Medium: ? O_2_: ? Shaking: ?	HeLa Medium: ? Incubation : 30 min or 3 h	MR (non-T1F-mediated) adhesion to HeLa cells was observed	Jones and Richardson, 1981
2.	Typhimurium	S6354, S6358, S1566, S850F (T1F+); S6351, S6352, S1566, S2204, S8x‘50 (T1F-)	Temp: 37°C Time: 4 SP, 48h Medium: tubes of 10 ml nutrient broth O_2_: aerobically Shaking: No	HEp-2, HeLa Medium: MEM + 0.5% FBS Incubation: 30 min or 90 min	MS adhesion of T1F+ strains	Tavendale et al., 1983
3.	Typhimurium	6354, 6358 (T1F+)	Temp: 37°C Time: 4 SP, 48h Medium: tubes of 10 ml nutrient broth O_2_: aerobically Shaking: No	HEp-2 Medium: MEM + 0.5% FBS Incubation: 30 min	MS adhesion	Old et al., 1986
4.	Typhimurium	SR-11 X3306	Temp: 37°C Time: grown till logarithmic growth phase Medium: brain heart infusion broth O_2_: Anaerobic growth in an atmosphere of 5% C02,10% H2, and 85% N2 (T1F+); aerobic growth was carried out in a gyratory shaker (T1F-) Shaking: ?	HEp-2 Medium: MEM Incubation: 3 h	MS adhesion of T1F+ strain	Ernst et al., 1990
5.	Typhimurium, Braenderup	Typhimurium- 501 (T1F+); 501NP, 503 (T1F-);Braenderup- 301, 302 (T1F+); 302NP, 303 (T1F-)	Temp: ? Time: overnight Medium: L-broth or L-agar plate O_2_: ? Shaking: ?	HeLa Medium: MEM Incubation: 2 h or 18 h	MS adhesion of T1F+ strain	Horiuchi et al., 1992
6.	Typhimurium	IR715, a derivative of ATCC 14028; SR-11 derivativeAJB3 (T1F+); AJB4 (Δ*fim*, T1F-)	Temp: ? Time: ? Medium: broth O_2_: aerobically Shaking: No	HEp-2, T-84, Int-407, HeLa, MDCK Medium: ? Incubation: ?	T1F-dependent adhesion to HeLa	Bäumler et al., 1996
7.	Enteritidis	S1400/94; LA5, 27655R	Temp: 37°C Time: 24h Medium: nutrient broth O_2_: aerobically Shaking: No	INT-407, Caco-2 Medium: EMEM+10% FBS Incubation: 2 h	T1F-dependent adhesion, Int-407- MS adhesion of T1F+ strain	Dibb-Fuller et al., 1999
8.	Typhimurium	SR-11 derivates- X 4252 (T1F+), 4253 (T1F-)	Temp: 37°C Time: untilbacteria were in mid-log phase growth Medium: LB O_2_: ? Shaking: Static	SI-H10, MM45T.BL Medium: ? Incubation: 1 h	T1F-dependent adhesion to SI-H10	Thankavel et al., 1999
9.	Enteritidis	-	Temp: 37°C Time: 48 to 72 h Medium: CFA or T O_2_: aerobically Shaking: No	HT-29, Caco-2 Medium: ? Incubation: ?	HT-29- T1F- strain invades better, Caco-2- T1F- strain invades equally,	Rajashekara et al., 2000
			Temp: 37°C Time: 24 h Medium: CFAbroth O_2_: aerobically Shaking: Yes (gentle shaking)	HD-11, MQ-NCSU Medium: Hank’s balanced salt solution Incubation: 45 min	No differences	
10.	Gallinarum; Pullorum; Typhimurium	297; 2933; LT2	Temp: 37°C Time: 48h Medium: Luria broth O_2_: aerobically Shaking: No	HEp-2 Medium: ? Incubation: ?	Presence of Typhimurium T1F increases adhesion and invasion rate of Gallinarum and Pullorum	Wilson et al., 2000
12.	Typhimurium, Enteritidis	Typhimurium- no information, Enteritidis- isolate no. 327	Temp: 37°C Time: 5 passages Medium: LB broth O_2_: ? Shaking: No	HT-29 Medium: a-MEM+10% FBS; Hu 1703He Medium: Fib41B+10% FBS; Incubation: 2 h	MS adhesion to cell lines	Kisiela et al., 2006
13.	Typhimurium	BJ2710- SL1344 derivative containing the LB5010 *fimH* gene (T1F+);BJ2508- BJ2710 *fimH::kan* (T1F-)	Temp: 37°C Time: 48 h Medium: 10 ml of LB broth O_2_: ? Shaking: No	HEp-2 Medium: RPMI+10% FBS Incubation: 24 hchicken intestinal epithelium Medium: RPMI+7% FBS+3%chicken serum Incubation: 24 h	T1F-dependent biofilm formation	Ledeboer et al., 2006
14.	Typhimurium	SL1344, ALB3, LB5010,Isogenic model with expression of FimH variants	Temp: 37°C Time: overnight Medium: SB O_2_: ? Shaking: No	HEp-2 Medium: ? Incubation: 1 h	FimH variant dependent adhesion	Kisiela et al., 2011
15.	Typhimurium	SL1344	Temp: ? Time: ? Medium: ? O_2_: ? Shaking: ?	HeLa Medium: DMEM+10% FBS Incubation: 10′, 12′	T1F-dependent adhesion, MS adhesion	Misselwitz et al., 2011
16.	Gallinarum	Gallinarum- isolate no. 589/02 (1); Δ*fimH* mutant (2); Gallinarum with Enteritidis FimH variant (3)	Temp: 37°C Time: passaged five times Medium: LB broth O_2_: ? Shaking: No	HT-29 Medium: ? Incubation: 2 h	1- No adhesion 2- MR adhesion 3- MS adhesion	Kuźmińska-Bajor et al., 2012
17.	22 serovars from subspecies I, 11 isolates from subspecies II-VII	Isogenic model with expression of FimH variants	Temp: ? Time: overnight Medium: ? O_2_: ? Shaking: ?	HEp-2, RAW264.7 Medium: ? Incubation: 1 h;	FimH variant dependent adhesion, MS adhesion	Kisiela et al., 2012
18.	Enteritidis	Isolate no. 327	Temp: 37°C Time: passaged five times Medium: LB broth O_2_: ? Shaking: No	ICE-1 Medium: IEC medium Incubation: 2 h	FimH dependent adhesion and invasion, MS adhesion and invasion	Kuźmińska-Bajor et al., 2015
19.	Choleraesuis	Isogenic model in Choleraesuis with expression of FimH variants	Temp: 37°C Time: passaged five times Medium: LB broth O_2_: ? Shaking: No	IPEC-J2 Medium: DMEM+10% FBS Incubation: 2 h	FimH variant dependent adhesion and invasion	Grzymajlo et al., 2017
20.	Typhimurium, Enteritidis, Gallinarum, Choleraesuis, Dublin	Isogenic model in Typhimurium with expression of FimH variants	Temp: 37°C Time: 48 h Medium: LB broth O_2_: ? Shaking: No	HEp-2,IPEC-J2 Medium: D-MEM/Ham’s F12 + 5% Incubation: 2 h	HEp-2- FimH variant dependent adhesion and invasionIPEC-J2- not-FimH- dependent adhesion and invasion	Kolenda et al., 2018


*Temp., Temperature; O_*2*_, presence of oxygen during growth; MR, mannose resistant; MS, mannose sensitive; T1F, type 1 fimbriae; “T1F+”, type 1 fimbriae expressing; “T1F-”, type 1 fimbriae non-expressing.*

The majority of studies using cell lines originating from intestinal epithelial cells, e.g., IPEC-J2, ICE-1, or HT-29, showed T1F-dependent and/or MS binding of *Salmonella* to cells (Kisiela et al., 2006; Kuźmińska-Bajor et al., 2012, 2015; Grzymajlo et al., 2017). There are also reports which showed no contribution of T1F to adhesion to these type of cells (Rajashekara et al., 2000; Kolenda et al., 2018). These conflicting results obtained with cell lines may stem from various experimental procedures during cultivation of bacteria, differences in adhesion assays or use of different *Salmonella* and non-fimbriate strains (Table 1).

Bacteria form biofilms to survive and persist within the host and environment (Flemming et al., 2016). Biofilm formation starts with adhesion to biotic and abiotic surfaces. It was shown that T1F are up-regulated during biofilm formation on cholesterol gallstones, but hyper fimbriation had a negative impact on biofilm formation. (Crawford et al., 2010; Gonzalez-Escobedo and Gunn, 2013). Moreover, T1F were found to contribute to biofilm formation on HEp-2 cells, murine and chicken intestinal epithelium, and plastic surfaces (Boddicker et al., 2002; Ledeboer et al., 2006).

## T1F and Animal Models

The role of T1F in *Salmonella* pathogenesis has been investigated using various animal models (20 studies was found, summarized in Table 2). The first such study on *S.* Typhimurium showed that a T1F+ strain was more infectious and virulent in mouse models than a non-fimbriated strain (Darekar and Duguid, 1972). In these studies, mice infected with T1F+ bacteria also excreted *S.* Typhimurium in their feces for a longer period of time. Similar results were obtained with follow-up experiments conducted by Duguid et al. (1976). A drawback of the aforementioned studies is that T1F- *Salmonella* strains used for testing were “natural” derivatives of T1F+ strains “induced” or “not-induced” for T1F production. Different results were obtained depending on the method used for generation of isogenic T1F- mutants of *S.* Typhimurium or *S.* Enteritidis, i.e., when transposon mutagenesis or the Datsenko-Wanner method were used. The work of Lockman and Curtiss (1992) revealed that a T1F-*S.* Typhimurium mutant was more virulent than the parental T1F+ strain. The authors hypothesized that the lower virulence of the T1F+ strain was due to sequestration of T1F+ bacteria in the liver, spleen and kidneys. The higher virulence of the T1F-*S.* Typhimurium strain in mouse infection models compared to the parental isogenic strain was further confirmed by van der Velden et al. (1998), who proposed that this phenomenon is caused by the expression of other virulence genes in the absence of T1F (van der Velden et al., 1998). Similar results were obtained with a *S.* Enteritidis Δ*fimH* mutant (Kuźmińska-Bajor et al., 2015). Using a mouse model, it was shown that the higher virulence of the T1F-*S.* Enteritidis compared to T1F+ bacteria could be attributed to higher systemic spread within the host. The authors suggested that T1F, which are responsible for adhesion to the intestinal mucosa, are a limiting factor in spreading bacteria outside of the intestinal tract. The direct involvement of T1F in the intestinal phase of infection was analyzed in a rat infection model, where expression of T1F in *S.* Enteritidis and *S.* Typhimurium adhering to enterocytes was demonstrated by immunohistochemistry (Ewen et al., 1997). However, long-term infection studies of rats challenged with the mixture of WT *S.* Enteritidis and a Δ*fimD* mutant revealed that the presence of T1F gives bacteria an advantage in the early stages of intestine infection, though higher counts of the T1F- strain were found in the intestine 6 days post infection (Naughton et al., 2001). In a 1-day old chicken model, the T1F-*S.* Enteritidis strain (which was also a mutant for the SEF14 and SEF17 fimbriae) had a lower ability than the *S.* Enteritidis WT strain to colonize the spleen, liver and caeca after 24 h of infection (Dibb-Fuller and Woodward, 2000). Similar results were obtained for *S.* Gallinarum in a study by Kuźmińska-Bajor et al. (2012), where a *fimH* deletion mutant had weak and delayed colonization of caecal tonsils, liver and spleen in comparison to its WT parent strain. In a study using laying hens, the T1F- strain of *S.* Enteritidis (Δ*fimD* deletion) was present in blood, caeca and oviducts for a longer time than the WT strain, though the WT strain was more frequently isolated from eggs (De Buck et al., 2004). On the other hand, T1F-*S.* Enteritidis (*fimA* single mutant) did not show any significant differences with the WT strain in colonization of the spleen and liver, and shedding of bacteria in feces of 5-day-old chickens (Rajashekara et al., 2000). The only difference found in the T1F- strain was a higher number of bacteria in the caecum 14 days post infection.

**Table 2:** Summary of animal experiments performed to study role of T1F in *Salmonella* virulence.

No.	Serovar	Strains	Growth conditions	Animal	Bacteria administration	Outcome	Source
1.	Typhimurium	1566F (T1F+); 1566N (T1F-)	Temp: 37°C Time: 24h Medium: nutrient broth O2: ? Shaking: ?	Mice- LAC Gray	Oral	Lowest proportion of mice infected and dying; shorter fecal shedding in comparison to oral; more successful infections and deaths with T1F+Non-fimbriated strain rapidly eliminated from the intestine,	Darekar and Duguid, 1972
					Conjuctival	Medium proportion of mice infected and dying; longer fecal shedding in comparison to oral; more successful infections and deaths with T1F+	
					intraperitoneal	Highest proportion of mice infected and dying; more deaths with T1F+	
2.	Typhimurium	1566F (T1F+); 1566N (T1F-)	Temp: 37°C Time: 24h Medium: Nutrient broth O2: Aerobically Shaking: No	Mice- LAC Gray	Oral	Higher number of infections and deaths in T1F+ strain than in T1F- strain	Duguid et al., 1976
					Conjuctival	Similar number of infections and deaths for T1F+ and T1F- strain	
					Intraperitoneal	Similar number of infections and deaths for T1F+ and T1F- strain	
3.	Enteritidis	1981	Temp: 37°C Time: ? Medium: Brain heart infusion O_2_: Aerated Shaking: No	The Naval Aero-Medical Reserve Unit (NAMRU) strainmice (12–14 weeks old)	Oral	Protective role of antiserum indicates that adherence of *S.* Enteritidis to the host cells was mediated by type 1 or by type 3 fimbriae	Aslanzadeh and Paulissen, 1990
4.	Typhimurium	SR-11 (T1F+) and x4334- *fim* transposon mutant (T1F-)	Temp: 37°C Time: ? Medium: Luria-Bertani or Mueller-Hinton O_2_: ? Shaking: No	BALB/c ♀ Mice (6–8 weeks old)	Oral	Higher virulence of T1F- strain (lower LD_50_ and higher mortality)	Lockman and Curtiss, 1992
					Intraperitoneal	No differences between tested strains	
					Oral- mixed bacteria challenge	Peyer’s patches, intestinal wall – higher amount of T1F- strainSpleen, liver, kidney – 2–3 days – higher amount of T1F- strainSpleen, liver, kidney- 4–5 day- higher amount of T1F+ strainBlood- higher amount of T1F- strain (increasing after each day	
5.	Enteritidis; Typhimurium	857, phage type 4 (SE)S986 (ST) (T1F+)	Temp: ? Time: 48 h Medium: Nutrient broth O_2_: ? Shaking: No	Male Hooded Lister rats	oral	T1F+ bacteria can be detected in ileum after 6 days of infection	Ewen et al., 1997
6.	Typhimurium	AJB3 ( SR11 derivate, T1F+); ABJ4 (T1F-)	Temp: 37°C Time: Overnight Medium: Luria-Bertani O_2_: ? Shaking: ?	BALB/c ♀ Mice (6–8 weeks old)	oral	T1F- strain is 3 times more virulent than T1F+ strain.	van der Velden et al., 1998
7.	Enteritidis	LA5 (T1F+); EAV21 (T1F-)	Temp: 37°C Time: Overnight Medium: Nutrient broth O_2_: Aerobically Shaking: Yes (Orbital shaking 225rpm)	SPF White Leghorn chicks (aged 18–24 h)		Collectively, SEF17, SEF21 and flagella fulfill a minor role in the early stages of colonization and invasion in young chicks, but are unnecessary for colonization of birds from the immediate environment	Dibb-Fuller and Woodward, 2000
8.	Enteritidis	phage type 4	Temp: ? Time: ? Medium: ? O_2_: ? Shaking: ?	SPF Chicken (SPAFAS Inc., Roanoke, IL)		No major role for SEF14, SEF17, or SEF21 fimbriae under the conditions tested.	Rajashekara et al., 2000
9.	Enteritidis	LA5	Temp: 37°C Time: 48 h Medium: Nutrient broth O_2_: ? Shaking: No	Male Hooded Lister rats (19 days old)		The fimbriate strain was preferentially removedfrom the gastrointestinal tract, allowing the mutantstrain to become predominant in the long term. Lower mount of T1F-strain in spleen after 24h; Lower mount of T1F-strain in liver after 24h; Lower mount of T1F-strain in caecum after 24h and 48h	Naughton et al., 2001
10.	Typhimurium	798-519’ (pig origin) (T1F+); Mutant 14 (ΔfimA; T1F-)	Temp: ? Time: ? Medium: ? O2: ? Shaking: ?	ICR mice (Harlan)	Oral-mixed bacteria challenge	Higher amounts of T1F+ strains in caecum, ileum and colon	Althouse et al., 2003
				BALB/c	Oral	No statistically significant results	
				Pigs;	Oral	Faster clearance of T1F- strain from ileocecal junction and mid-ileum. T1F+ strain was recovered in increasing numbers after 2 weeks in comparison to 1 week after challenge	
11.	Enteritidis	S1400/94 (T1F+); ΔfimD of S1400/94 (T1F-)	Temp: 37°C Time: 20 h Medium: Brain Heart Infusion O2: ? Shaking: Yes	non-Salmonella-vaccinated laying hens (ISA Warren Brown) (19 weeks old)	Intravenous	Higher amounts of T1F- strains in spleen after 14 and 21 daysHigher amounts of birds positive for T1F- strains in swabs: from vagina and isthmus after 21 days. Higher amounts of egg shells contaminated with T1F+.	De Buck et al., 2004
12.	Enteritidis	??	Temp: ? Time: ? Medium: ? O_2_: ? Shaking: ?	SHAVER 579 hens (5 weeks old) 1 group was immunized twice with FimA protein	Oral	significant reduction of duodenum colonization and persistence of *Salmonella* Enteritidis	Kuczkowski et al., 2004
13.	Enteritidis	phage type 4, strain S1400/94; strain MB 1454	Temp: 37°C Time: 20 h Medium: Brain Heart Infusion O_2_: ? Shaking: Yes	non-*Salmonella*-vaccinated laying hens (ISA Warren Brown) (18 weeks old); 1 group was vaccinated with twice with purified T1F	Intravenous	Higher amounts of egg shells contaminated in non-vaccinated chickens. Higher and longer colonization of oviducts in non-vaccinated chickens.	De Buck et al., 2005
14.	Typhimurium	1402/84 (ClinicalIsolate)	Temp: 37 °C Time: 60 h Medium: Colonizationfactor antigen (CFA) agar O_2_: ? Shaking: ?	Male albino Wistar rats (50–60 g); One group of rats was immunized with purified T1F	Oral	Immunization prevents from: Na^+^, Cl^-^, Ca^2+^ fluxes in intestines; cAMP, Prostaglandin E2 concentration changes in intestines; NADPH, G-6-PDH 6-PGDH changes in gut macrophages	Verma et al., 2005
15.	Enteritidis	phage type 4, strain P125109 (T1F+); Δ*fimA* of P125109 (T1F-)	Temp: ? Time: ? Medium: LB O_2_: ? Shaking: ?	SPF out-bred Rhode Island Red chickens, 18-day-old	Oral	No significant changes in caecal load after 3, 7, 10 days post-infection	Clayton et al., 2008
16.	Gallinarum	isolate no. 589/02 (1,T1F+); Δ*fimH* of 589/02 (2, T1F-); 589/02 with *fimH* gene from *S.* Enteritidis (3, T1F+)	Temp: 37°C Time: five passages Medium: Luria-Bertani O_2_: ? Shaking: No	Salmonella-free chicks (1-day-old)	Oral	FimH-dependent interactions of *S.* Gallinarum with chicken leukocytes are responsible for the increased virulence in chicksT1F- strain (2) had weak and delayed colonization of caecal tonsils, liver and spleen, didn’t colonize bursa of Fabricius. T1F+ strain (3) didn’t colonize caecal tonsils, bursa of Fabricius, liver had weak and delayed colonization of spleen.	Kuźmińska-Bajor et al., 2012
17.	Typhimurium	SL1344 expressing various FimH variants	Temp: ? Time: Overnight Medium: SB broth supplemented with 30 mg/ml chloramphenicol O_2_: ? Shaking: No	BALB/c mice (6–8 week-old)	Oral	No effect of FimH mutations on bacterial burdens inthe liver and spleen.	Kisiela et al., 2012
18.	Enteritidis	JL12	Temp: 37°C Time: 72h Medium: Colonization factor antigen broth O_2_: ? Shaking: No	Salmonella-free Hy-Line white leghorn chickens; (1 day old); one group was immunized twice orally with liposome associated SEF21 gene		Lower amounts of *Salmonella* in cecum and rectum after 4 weeks in immunized bacteria	Pang et al., 2012
19.	Enteritidis	SD-2	Temp: ? Time: ? Medium: ? O_2_: ? Shaking: ?	BALB/c mice; Groups of mice were immunized with FimA protein and it’s derivates with mC3d extenstions	Intraperitoneal	Lower infection of mice immunized with FimA proteins constructs	Musa et al., 2014
20.	Enteritidis	isolate no. 327 (T1F+); Δ*fimH* of 327 (T1F-)	Temp: 37°C Time: Five passages Medium: Luria- Bertani broth O_2_: ? Shaking: No	BALB/c ♀ Mice (6–8 weeks old)	oral	Fimbriated wild-type *S.* Enteritidis is less virulent than the non-fimbriated *S.* Enteritidis mutant strain	Kuźmińska-Bajor et al., 2015


*Temp., Temperature; O_*2*_, presence of oxygen during growth; T1F, type 1 fimbriae; “T1F+”, type 1 fimbriae expressing; “T1F-”, type 1 fimbriae non-expressing; “SPF”, specific pathogen free.*

## Receptors for T1F

The discovery of T1F-dependent, MS agglutination of yeast and guinea pig RBCs led to the hypothesis that oligosaccharide chains containing mannose residues are receptors for T1F. Therefore, the carbohydrate specificity of *S.* Typhimurium T1F was analyzed by inhibition of agglutination with linear and branched mannose-containing oligosaccharides or glycosides of D-mannose. It was found that binding of T1F+ *S.* Typhimurium to yeast cells and guinea pig RBCs was inhibited most efficiently by high mannose oligosaccharides (Firon et al., 1983, 1984).

One of the intriguing issues concerning *Salmonella* T1F is the innate T1F receptors expressed at the surface of host cells. Leusch et al. (1991) analyzed binding of various *Salmonella* serovars to glycoproteins expressed in the intestine, egg white, blood, spleen and bile. It was found that most of the *Salmonella* Typhi, Paratyphi A and B, and Java isolates bound to carcinoembryonic antigen (CEA). It was also revealed, that *S.* Typhi bound with the highest affinity to CEAs and an unknown glycoprotein, NCA-55. However, MS binding was only tested and shown for adhesion of *S.* Typhi to CEA. In another study, an extracellular matrix protein, laminin, was found as a receptor for *S.* Enteritidis and *S.* Typhimurium T1F, and the glycan part of laminin was bound by T1F in a MS manner (Kukkonen et al., 1993). Another possible receptor for *S.* Typhimurium T1F is a 60-kDa glycoprotein that was isolated from the brush border of a rat’s small intestine, but the protein was not further characterized (Ghosh et al., 1996). This glycoprotein interacted with isolated T1F in MS manner. *S.* Enteritidis T1F receptor, responsible for infection of chicken eggs during egg production, was found in the isthmus of the chicken reproductive tract. Binding of T1F+ *S.* Enteritidis to isthmus sections and secretions were blocked by mannose and mediated by mannosylated glycoproteins, which were detected in isthmus using lectins (De Buck et al., 2003). The best characterized *Salmonella* T1F receptor to date is pancreatic secretory granule membrane major glycoprotein GP2, first identified as a transcytotic receptor of M cells for T1F+ *S.* Typhimurium in human and mice. Translocation of FimH-positive *S.* Typhimurium through M cells leads to increased numbers of bacteria in the mesenteric lymph nodes and the immune response to antigens expressed by these bacteria (Hase et al., 2009). The adhesion of *S.* Typhimurium expressing FimH variants from serovars Typhimurium, Enteritidis, Dublin and Choleraesuis to porcine GP2 was shown recently (Kolenda et al., 2018).

A study on binding of *S.* Enteritidis, *Salmonella* Choleraesuis, *Salmonella* Dublin and *S*. Abortus-ovis FimH proteins to cell lysates from intestinal cell lines originating from various potential hosts (pig, sheep and cow) revealed that FimH binds in an MS manner to different glycoproteins, depending on serovar host range. FimH from generalist *S.* Enteritidis bound to surface membrane proteins of about 130 kDa, while FimH from host specialists bound to a protein of about 55 kDa (Grzymajło et al., 2013). A recent study identified a 55 kDa receptor as calreticulin (CRT; Grzymajlo et al., 2017). It was shown that CRT isolated from porcine IPEC-2 cells was bound specifically by *S.* Choleraesuis FimH and not by FimH from *S.* Enteritidis, suggesting that host-specificity of *Salmonella* serovars is dependent on both pathogen and host factors.

Glycosphingolipids are common components of the plasma membrane of cells. Studies by Li et al. (2003a,b) revealed that glucosylceramide (GlcCer) and monosialodihexosylganglioside (GM3) are possible receptors for *S.* Enteritidis T1F (Li et al., 2003a,b). The binding of GlcCer and GM3 isolated from intestinal mucosa and chicken oviductal tracts bound to T1F-expressing *S.* Enteritidis and this binding was blocked by anti-T1F antibodies. The only non-glycan-mediated binding of T1F was shown in *S.* Typhimurium, which bound to plasminogen (Kukkonen et al., 1998). This interaction was blocked by a lysine analog and not mannose.

## Role of *FimH* Allelic Variation in Pathogenesis

**FIGURE 3:**
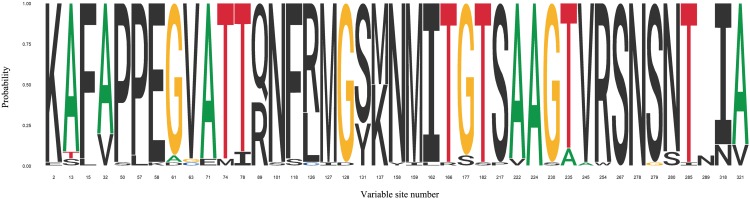
Sequence logo for FimH variable sites identified in 29 *Salmonella* serovars. FimH variable sites were extracted from articles discussed in this review. Detailed information about serovars can be found in Supplementary Table 1.

FimH adhesin located at the top of T1F is directly involved in binding to different high-mannose oligosaccharides carried by surface glycoproteins of eukaryotic cells. Despite being very high, reaching 99% sequence homology, it became increasingly clear that significant micro-heterogeneity, associated with differences in the amino acid sequences of FimH adhesins, exists among type 1 fimbriae from different serovars and affects their binding to mannosylated oligosaccharides (Figure 3 and Supplementary Table 2).

The discovery that *fimH* allelic variants found in two *S.* Typhimurium strains (LB5010 and SL1344), which differ in amino acid at positions 61 and 118, are responsible for different adhesion phenotypes, started a new period in T1F studies (Boddicker et al., 2002). The FimH variant from strain SL1344, with glycine and phenylalanine at positions 61 and 118, respectively, mediated low binding of *Salmonella* to HEp-2 cells and the FimH variant from strain LB5010, with alanine and serine at positions 61 and 118, respectively, mediated a high level of *Salmonella* binding to the same cells. When the amino acid sequence of *Salmonella* Enteritidis FimH was compared with the low-binding variant of *S.* Typhimurium FimH adhesin, it was found that *S.* Enteritidis FimH also represent the low-binding phenotype with glycine in position 61 and phenylalanine in position 118 (Kisiela et al., 2006). However, follow-up experiments revealed that only the substitution from phenylalanine to serine at position 118 contributes to a change from the low to high binding phenotype, at least in the case of *S.* Enteritidis (Grzymajlo et al., 2010). It has been previously mentioned that T1F mediate binding of *Salmonella* to HEp-2 cells. Substitution at position 158 from asparagine to tyrosine within the *S.* Typhimurium FimH increases adhesion of bacteria to HEp-2 cells and DCs (Guo et al., 2009). Screening of various *Salmonella* serovars for FimH variation and MS adherence to HEp-2 cells, and biofilm formation further confirmed the role of sequence variability in the binding properties of *Salmonella* FimHs (Dwyer et al., 2011). The magnum opus on the association with and the role of *fimH* allelic variants in the pathogenesis of salmonellosis caused by *Salmonella* serovars with different host ranges was published by Kisiela et al. (2012). The authors investigated the effects of sequence variation on binding of FimH adhesins to purified high-mannose type glycoproteins, epithelial and macrophage-like cell lines. Based on these results, FimH high- and non-binding variants were linked with host specialists (host-restricted and host-adapted serovars) and FimH low-binding variants were linked with host generalists (host-unrestricted serovars). However, in mouse infection models no significant differences in colonization of spleen and liver were observed for *S.* Typhimurium isogenic strains expressing FimH variants representing high-, low- or non-binding phenotypes, and no contribution of FimH variation to disease phenotype was found. In a more recent study, Yue et al. (2015) searched for the presence of FimH variants among *S.* Typhimurium strains isolated from human and bovine hosts and analyzed host-specific adhesion to human and bovine cell lines (Yue et al., 2015). Sequence analysis of *fimH* from 580 isolates revealed that the presence of valine residue at position 245 was found more often in human isolates and alanine at the same position was found more often in bovine isolates. An adhesion assay showed that substitution from valine to alanine at position 245 in FimH from *S.* Typhimurium increased binding to cells of bovine origin without affecting binding to cells of human origin. This study also showed that FimH variants from *Salmonella* specialists mediated binding to cell lines in a host-specific manner. For example, FimH from swine-associated *S.* Typhisuis and *S.* Choleraesuis serovars bound better to porcine IPEC-1 and IPEC-J2 cells, whereas FimH from human-associated *S.* Typhi and *S.* Newport isolates of human origin bound better to human RKO, HCT116 and Caco-2 cells. What is more, FimH from bovine-associated *S.* Dublin isolates bound better to bovine C8 and CMS cells, and FimH from poultry-associated *S.* Gallinarum isolates bound better to avian LMH cells than to all other aforementioned cells. In another study, Schifferli’s group analyzed the role of FimH allelic variants in adhesion of *S.* Newport strains isolated from bovine, porcine and human hosts to cell lines of the same host origin (De Masi et al., 2017). It was shown that strains of bovine and porcine origin, carrying a FimH variant with phenylalanine at position 15, alanine at position 32 and arginine at position 89 bound better to cell lines of bovine (CMS, J8) and porcine origin (IPEC-J2) than to human cell lines. On the other hand, *Salmonella* strains of human origin, carrying a FimH variant with leucine at position 15, valine at position 32 and glutamine at position 89 bound better to human cell lines (RKO, Caco-2) than to bovine or swine cell lines.

Studies employing random mutagenesis and 3D structure predictions of the FimH from *S.* Typhimurium showed that mutations in the predicted lectin domain, the interdomain and the pilin domain can lead to a change in FimH binding properties. It was proposed that binding of FimH to its receptor under shear force leads to activation of allosteric properties in FimH variants, which can alter the binding properties of these FimH variants compared to their binding under static conditions (Kisiela et al., 2011). For example, a FimH variant from *S.* Typhimurium SL1344 exhibited a higher degree of binding to the same glycoproteins when tested under shear conditions than in a static adhesion assay. On the other hand, FimH variants that had higher binding properties in static conditions had weaker binding under shear stress.

The GP2 of both human and mouse was shown to be the receptor for T1F of *S.* Typhimurium (Hase et al., 2009). The influence of *fimH* variation on binding to human and porcine GP2 isoforms expressed in SF9 cells was tested in a study by Kolenda et al. (2018). The authors used FimH variants from five *Salmonella* serovars and found that binding to different GP2 isoforms was FimH variant-dependent and not GP2-host origin-dependent or GP2-variant-dependent. Another example of an association of FimH variation with recognition of host proteins can be found in the previously described study by Grzymajło et al. (2013), which identified substitutions in positions L57P and N101S in FimH as altering receptor specificity and possibly contributing to changes in host range of *Salmonella* serovars.

Lee and Yeh observed that T1F production in *S.* Choleraesuis is dependent on amino acid variation at residue 63. They analyzed the expression of T1F in *S.* Choleraesuis, showing that only 4 out of 120 strains expressed T1F (Lee and Yeh, 2016). All strains expressing T1F had a valine residue at position 63 and strains without T1F expression had glycine at the same position.

Fimbriae of serovars not agglutinating RBCs and yeast cells (MR T1F), i.e., *Salmonella* Gallinarum and *S*. Paratyphi B, were initially called type 2 fimbriae, but genetic and microscopic analysis showed that these fimbriae were T1F (Crichton et al., 1989; Kisiela et al., 2005). In the case of *S.* Gallinarum T1F, the loss of MS binding was linked to a single amino acid substitution, from threonine to isoleucine, at position 78 of FimH (Kisiela et al., 2005). Whether the MR phenotype of *S.* Gallinarum FimH confers the inactive T1F phenotype because T1F is unable to bind to any receptors or whether the MR phenotype leads to changes in receptor specificity was investigated by Guo et al. (2009) who provided proof that the latter is, in fact, true by demonstrating that *S.* Gallinarum T1F mediates binding to chicken leukocytes. Another study investigating the role of FimH variation in the pathogenesis of *S.* Gallinarum showed that expression of MS *S.* Enteritidis FimH in *S.* Gallinarum leads to decreased or no colonization of liver, spleen and caecal tonsils, thus proving a significant role for FimH variation in bacterial host specificity (Kuźmińska-Bajor et al., 2012). Investigations by Guo et al. (2009) and Kuźmińska-Bajor et al. (2012) revealed that T1F variation can confer a significant advantage for *Salmonella* Gallinarum during pathogenesis in chickens, an observation that could explain such a drastic change like the switch from MS to MR phenotype.

## Future Prospects

In this article, we have presented both early and recent studies that describe the importance of T1F in *Salmonella* infections. During the last 60 years, investigations have been carried out to establish the role of T1F in *Salmonella* pathogenesis. Although much has been revealed about the functions of T1F, many unanswered questions remain to be addressed in future studies.

T1F mediate binding to host tissues, in which FimH is directly involved, is one of the first steps of *Salmonella* pathogenesis. Designing or finding inhibitors that can block the binding of T1F to receptors could provide new options for prevention and treatment of *Salmonella* infections. Unfortunately, the crystal structure of FimH from *Salmonella* is yet to be resolved, which significantly hampers structure-based inhibitor design and the search for new inhibitors. Design of new T1F inhibitors could be also aided by more complete identification and characterization of receptors expressed on host tissues. While some studies have reported proteins binding with T1F, knowledge of the range of receptors present in *Salmonella* serovars other than *S.* Typhimurium and *S.* Choleraesuis is still limited. Moreover, already identified receptors, such as CRT and GP2, require further research to study interactions between FimH and glycosylation as well as to describe the accessibility and expression of these receptors in host organs.

Although binding of T1F+ *Salmonella* to intestines has been shown, there are no studies about spatial expression of T1F within the host during *Salmonella* infection. Such studies would allow researchers to more fully elucidate where and for how long T1F are expressed, which could improve the search for T1F receptors by allowing efforts to focus on host sites directly involved in T1F-mediated *Salmonella* adhesion. It was proposed that T1F might have different functions in the intestinal phase of *Salmonella* infection in host specialists and generalists. Taking into account the recent developments in microbiomics and the importance of inflammation during *Salmonella* gastroenteritis, it would be interesting to investigate how or if T1F contribute to the development of inflammatory diarrhea caused by *Salmonella* generalists and whether the host range associated SNPs in FimH can alter infection outcomes.

Advances in next generation sequencing (NGS) technologies have resulted in the availability of thousands of *Salmonella* genomes, providing a great opportunity to study the genetic organization of the *fim* gene clusters present in many *Salmonella* serovars, representing different host specialists and host generalists. NGS data can be used to assess variability in T1F coding regions and to explore how that variability translates into T1F regulation, expression, and host specific actions. NGS also brings new tools for analyzing regulation of T1F in *Salmonella.* As a large part of our review recapitulates regulation of T1F expression in *Salmonella* (mainly *S.* Typhimurium), it is clear that there is still a considerable amount work to be done in this area, in particular there is a need for a systematic assessment of T1F expression *in vitro* and *in vivo*. One approach to achieving this would be to prepare a mutant library of the *fim* gene cluster of one *Salmonella* host generalist and specialist (e.g., *S.* Typhimurium or *S.* Enteritidis and *S.* Typhi or *S.* Gallinarum) and compare the influence of particular mutations on expression of T1F and on crosstalk between T1F and other virulence factors. Use of NGS could also facilitate studies of spatial expression of T1F *in vivo* and assessment of immune responses during host infection.

The role of *fimH* allelic variation on expression of T1F and other virulence factors is another interesting topic that requires further research. A study by Lee and Yeh (2016) showed that a single SNP (V63G) in *fimH* is responsible for a lack of T1F expression in *S.* Choleraesuis. However, it should be noted that this result does not accord with our observations of expression of the same FimH variant in a WT *S.* Choleraesuis isolate, in isogenic (V63G) model generated in another *S.* Choleraesuis isolate (Grzymajlo et al., 2017). A deleterious effect of the V63G mutation was observed on FimH expression in *S.* Typhimurium SL1344, but the same variant was expressed in one *S.* Choleraesuis isolate in a study by Kisiela et al. (2012). Two isogenic models generated for expression of different FimH variants in *S.* Typhimurium (Kisiela et al., 2012; Kolenda, 2018) showed differences in T1F expression, although it must be noted that only one antibody was used to assess T1F expression. These data indicate that there is a phenotypic variation in T1F expression in *Salmonella* that is associated with SNPs in FimH sequence as well as other unknown factors. It is possible that lack of T1F expression results in increased expression of other virulence factors that are beneficial for successful host infection.

## Author Contributions

RK and KG conceptualized, wrote original draft, visualized, and prepared the figures and tables. MU wrote the review and edited the manuscript. All authors discussed the results and contributed to the final manuscript.

## Conflict of Interest Statement

The authors declare that the research was conducted in the absence of any commercial or financial relationships that could be construed as a potential conflict of interest.
