# Supplementary material for: Everything You Always Wanted to Know About Salmonella Type 1 Fimbriae, but Were Afraid to Ask
Source: Front Microbiol. 2019 May 14;10:1017. doi: 10.3389/fmicb.2019.01017 (PMC6527747; doi:10.3389/fmicb.2019.01017)
Supplement: Supplementary file 1 [file Table_1.DOCX]

| No. | Year | Authors | Title | Serovar | Strains | Growth conditions | Cells | Outcome |
| --- | --- | --- | --- | --- | --- | --- | --- | --- |
| 1. | 1958 | JP Duguid, RR Gillies | Fimbriae and haemagglutinating activity in *Salmonella*, *Klebsiella, Proteus* and *Chromobacterium* | 18 serovars | 81 isolates | Temp: 37°C  Time: serial cultivation for 48 h  Medium: broth?  O_2_ aerobically  Shaking: ? | Guinea-pig, horse, chicken erythrocytes | Strong MS agglutination of T1F+ strains |
|  |  |  |  |  |  |  | Rabbit, human erythrocytes | Weak MS agglutination of T1F+ strains |
|  |  |  |  |  |  |  | Sheep, ox erythrocytes | Very weak or no MS agglutination of T1F+ strains |
| 2. | 1966 | JP Duguid, ES Anderson, I Campbell | Fimbriae and adhesive properties in *Salmonellae* | 157 serovars | 1453 isolates | Temp: 37°C or 30°C  Time: serial cultivation for 48 h  Medium: broth?  O_2_: aerobically  Shaking: ? | Guinea-pig erythrocytes | Agglutination in case of 1138 isolates,  MS agglutination- only selected isolated were tested |
| 3. | 1968 | DC Old, I Corneil, LF Gibson, AD Thomson, JP Duguid | Fimbriation, pellicle formation and the amount of growth of *Salmonellas* in broth. | Typhimurium, Choleraesuis, Paratyphi B, | 22 isolates | Temp: 37°C  Time: serial cultivation for 48 h  Medium: broth?  O_2_: aerobically  Shaking: static | Guinea-pig erythrocytes | Agglutination of T1F+ strains |
| 4. | 1969 | JP Duguid, I Campbell | Antigens Of The Type-1 Fimbriae Of Salmonellae And Other Enterobacteria | Multiple serovars | - | Temp: 37°C  Time: serial cultivation for 48 h  Medium: broth?  O_2_: aerobically  Shaking: static | Guinea-pig erythrocytes | Agglutination of T1F+ strains |
| 5. | 1970 | DC Old, SB Payne | Antigens Of The Type-2 Fimbriae Of Salmonellae Cross-Reacting Material (Crm) Of Type-1 Fimbriae | Paratyphi B, Pullorum | S543, S859, S867, S1076, S2416 (Group 1); S66, S957, S298, S318, S1662 (Group 2) | Temp: 37°C  Time: serial cultivation for 48 h  Medium: 10ml of nutrient broth  O_2_: aerobically  Shaking: static | Guinea-pig erythrocytes | Group 1- agglutination of T1F+ strains;  Group 2- no agglutination of T1F+ strains |
| 6. | 1970 | DC Old, JP Duguid | Selective outgrowth of fimbriate bacteria in static liquid medium. | Typhimurium | 14 isolates | Temp: 37°C  Time: serial cultivation for 48 h  Medium: 10-ml volumes  of the phosphate-buffered broth  O_2_: aerobically/anaerobically  Shaking: static | Guinea-pig erythrocytes | Agglutination of T1F+ strains |
| 7. | 1975 | JP Duguid, ES Anderson, GA Alfredsson, R Barker, DC Old | A new biotyping scheme for *Salmonella* typhimurium and its phylogenetic significance. | Typhimurium | 2030 isolates | Temp: 37°C  Time: serial cultivation for 48 h  Medium: 10ml of nutrient broth  O_2_: aerobically  Shaking: static | Guinea-pig erythrocytes | Agglutination in case of 1711 isolates |
| 8. | 1975 | GW Tannock, RVH Blumershine, DC Savage | Association of Salmonella typhimurium with, and its invasion of, the ileal mucosa in mice. | Typhimurium | LT2, TV119, G30, SL1032 | Temp: 30°C  Time: serial cultivation  Medium: brain heart infusion broth  O_2_: ?  Shaking: ? | Guinea-pig erythrocytes | Agglutination of all tested isolates |
| 9. | 1976 | JP Duguid, MR Darekar, DWF Wheater | Fimbriae and infectivity in *Salmonella* Typhimurium | Typhimurium | Strain no. 1566 | Temp: 37°C  Time: ?  Medium: nutrient broth  O_2_: aerobically  Shaking: static | Guinea-pig erythrocytes | Agglutination of T1F+ strains |
| 10. | 1981 | TK Korhonen, H Leffler, C Svanborg Eden | Binding specificity of piliated strains of *Escherichia coli* and *Salmonella typhimurium* to Epithelial Cells, *Saccharomyces cerevisiae* Cells, and Erythrocytes. | Typhimurium | SH6749 (a nonflagellated *galE*  mutant of the LT2 line) | Temp: ?  Time: ?  Medium: broth?  O_2_:?  Shaking: ? | *Saccharomyces cerevisiae* | MS agglutination |
|  |  |  |  |  |  |  | Human urinary epithelial cells | MS adhesion |
|  |  |  |  |  |  |  | Human buccal epithelial cells | No adhesion |
|  |  |  |  |  |  |  | Rat urinary epithelial cells | MS adhesion |
| 11. | 1981 | GW Jones, LA Richardson | The Attachment to, and Invasion of HeLa Cells by *Salmonella*  *typhimurium*: The Contribution of Mannose-sensitive and Mannose-resistant Haemagglutinating Activities | Typhimurium | TML, W118, NY, PR (T1F+); S850, S2204 (T1F-) | Temp: ?  Time: ?  Medium: broth?  O_2_:?  Shaking: ? | Guinea-pig, horse, chicken sheep erythrocytes | MS agglutination of T1F+ strains |
|  |  |  |  |  |  |  | Sheep, cow, human erythrocytes | MS agglutination of T1F+ strains(but lower when compared with other erythrocytes) |
| 12. | 1982 | RD Leunk, RJ Moon | Association of type 1 pili with the ability of livers to clear *Salmonella typhimurium* | Typhimurium | SR-11 (T1F+), SR-11 (T1F-, nonpiliated variant) | Temp: ?  Time: ?  Medium: broth?  O_2_:?  Shaking: ? | Guinea pig erythrocytes | MS agglutination of T1F+ strain |
| 13. | 1983 | N Firon, I Ofek, N Sharon | Carbohydrate specificity of the surface lectins of *Escherichia coli, Klebsiella pneumoniae*, and *Salmonella typhimurium*. | Typhimurium | from the collection  of the Department of Human Microbiology (Tel-Aviv University). | Temp: 37°C  Time: 48 h; SP until l0^7^ cells/mL gave a rate  of aggregation of 15-20 units/min.  Medium: 10ml of nutrient broth  O_2_: aerobically  Shaking: static | *Saccharomyces cerevisiae* | MS agglutination |
| 14. | 1983 | A Tavendale, KH Jardine, DC Old, JP Duguid | Haemagglutinins and adhesion of *Salmonella typhimurium* to HEp2 and HeLa cells | Typhimurium | S6354, S6358, S1566, S850F (T1F+); S6351, S6352, S1566, S2204, S850 (T1F-) | Temp: 37°C  Time: 48 h; 4SP  Medium: 10ml of nutrient broth  O_2_: aerobically  Shaking: static | Chicken, guinea-pig, horse, man (group 0), ox, pig and sheep erythrocytes | MS agglutination of T1F+ strains |
| 15. | 1984 | N Firon, I Ofek, N Sharon | Carbohydrate-binding sites of the mannose-specific fimbrial lectins of enterobacteria. | Typhimurium, Virginia, Tel Aviv, Emeck, Enteritidis, Paratyphi C | Paratyphi C was a fecal isolate; the other serotypes  were from the National Center of Enterobacteriaceae,  Government Central Laboratories, Jerusalem,  Israel. | Temp: 37°C  Time: 48 h; SP until l0^7^ cells/mL gave a rate  of aggregation of 15-20 units/min.  Medium: 10ml of nutrient broth  O_2_: ?  Shaking: static | *Saccharomyces cerevisiae* | MS agglutination |
| 16. | 1986 | DC Old, AI Roy, A Tavendale | Differences in adhesiveness among type 1 fimbriate strains of *Enterobacteriaceae* revealed by an in vitro HEp2 cell adhesion model | Typhimurium | 6354, 6358 (T1F+) | Temp: 37°C  Time: 48 h; SP (usually 5)  Medium: 10ml of nutrient broth  O_2_: aerobically  Shaking: static | Guinea-pig erythrocytes | MS agglutination |
| 17. | 1987 | BL Lindquist. E Lebenthal, P-C Lee, MW Stinson, JM Merrick | Adherence of *Salmonella typhimurium* to small-intestinal enterocytes of the rat. | Typhimurium | S74714ϕF, LT2, S850/PR22, CR660, AA2202, LT2 (Δ712) (T1F+); S7471N, CR8500 (T1F-) | Temp: 37°C  Time: ?  Medium: 10ml of LB  O_2_: aerobically  Shaking: static) | Rat, guinea pig, human  erythrocytes. | MS agglutination of T1F+ strains |
|  |  |  |  |  |  | Temp: 37°C  Time: 36 h;  Medium: 10 ml of LB in the presence of [8-3H]adenine (10 µCi/ml  O_2_: aerobically  Shaking: static | Rat enterocytes | Adhesion of T1F+ strains,  MS adhesion of S74714ϕF strains |
| 18. | 1989 | PB Crichton DE Yakubu, DC Old, S. Clegg | Immunological and genetical relatedness of type-1 and type-2 fimbriae in salmonellas of serotypes Gallinarum, Pullorum and Typhimurium. | Gallinarum, Pullorum,  Typhimurium | S615, S1172, LT2, S625F | Temp: 37°C  Time: 24 h;  Medium: 10-ml of phosphate-buffered broth in cotton wool-stoppered test tubes  O_2_: aerobically  Shaking: gentle shaking (100 rev/min)  rev/min) | Guinea-pig erythrocytes,  *Saccharomyces cerevisiae* | Tym- MS agglutination  Gall, Pull- no agglutination |
| 19. | 1989 | BA Oyofo, RE. Droleskey, JO Norman, HH Mollenhauer,  RL Ziprin, DE Corrier, JR DeLoach | Inhibition by mannose of in vitro colonization of chicken small intestine by *Salmonella typhimurium*. | Typhimurium | ST-10, ST-11(T1F+); Thax-1, ST-12 (T1F-) | Temp: 37°C  Time: overnight;  Medium: 5 mL of trypticase soy broth  O_2_: aerobically  Shaking: static | *Candida albicans* | MS agglutination of T1F+ strains |
| 20. | 1990 | J Aslanzadeh, LJ Paulissen | Adherence and pathogenesis of *Salmonella enteritidis* in mice. | Enteritidis | 1891 | Temp: 37°C  Time: 24 h;  Medium: Brain heart  infusion broth  O_2_: ?  Shaking: static  . | Horse, guinea pig, rabbit, rat, mouse,  chicken, sheep, goat, human erythrocytes | MS agglutination |
|  |  |  |  |  |  |  | Human buccal epithelial cells | MS adhesion |
|  |  |  |  |  |  |  | Mouse small intestine epithelial cells | MS adhesion |
| 21. | 1991 | HG Leusch, Z Drzeniek, Z Markos-Pusztai, C Wagener | Binding of *Escherichia coli* and *Salmonella* strains to members of the carcinoembryonic antigen family differential binding inhibition by aromatic alpha-glycosides of mannose. | Typhi, Paratyphi A, Paratyphi B, Java | - | Temp: ?  Time: ?  Medium: Luria broth.  O_2_: ?  Shaking: ? | Guinea-pig erythrocytes | MS agglutination of T1F+ strains |
| 22. | 1991 | KH Muller. SK Collinson, TJ Trust, WW Kay | Type 1 fimbriae of *Salmonella enteritidis*. | Enteritidis | 27655 | Temp: ?  Time: ?  Medium: liquid colonization  factor antigen medium supplemented with 5 mM KH_2_PO_4_ and 12 mM Na_2_HPO_4_ or in Luria broth  O_2_: ?  Shaking: static | Guinea-pig erythrocytes | MS agglutination |
| 23. | 1992 | HA Lockman, R Curtiss, III | Isolation and characterization of conditional adherent and non-type 1 fimbriated *Salmonella typhimurium* mutants | Typhimurium | LT2 WT and various mutants | Temp: ?  Time: ?  Medium: MH broth  O_2_: ?  Shaking: static | Guinea-pig erythrocytes,  *Saccharomyces cerevisiae* | MS agglutination of T1F+ strains |
| 24. | 1992 | J Aslanzadeh, LJ Paulissen | Role of type 1 and type 3 fimbriae on the adherence and pathogenesis of Salmonella enteritidis in mice. | Enteritidis | 1891-V and 1891-A | Temp: 37°C  Time: 48 h;  Medium: Brain heart  infusion broth  O_2_: ?  Shaking: static | Horse, guinea pig, rabbit, rat, mouse,  chicken, sheep, goat, human erythrocytes,  *Saccharomyces cerevisiae* | MS agglutination |
|  |  |  |  |  |  |  | Human buccal epithelial cells,  Mouse small intestine epithelial cells | MS adhesion |
| 25. | 1992 | S Horiuchi, Y Inagaki, N Okamura, R Nakaya, N Yamamoto | Type 1 pili enhance the invasion of *Salmonella braenderup* and *Salmonella typhimurium* to HeLa cells. | Typhimurium, Braenderup | Typhimurium- 501 (T1F+); 501NP, 503 (T1F-);  Braenderup- 301, 302 (T1F+); 302NP, 303 (T1F-) | Temp: ?  Time: overnight;  Medium: L-broth or L-agar plate  O_2_: ?  Shaking: ? | Guinea-pig erythrocytes | MS agglutination of T1F+ strains |
| 26. | 1992 | HA Lockman, R Curtiss III | Virulence of non-type 1-fimbriated and nonfimbriated nonflagellated *Salmonella typhimurium* mutants in murine typhoid fever | Typhimurium | isogenic derivatives of  SR-11 | Temp: 37°C  Time: ?  Medium: L broth or in Mueller-Hinton broth O_2_: ?  Shaking: static | Guinea-pig erythrocytes,  *Saccharomyces cerevisiae* | MS agglutination of T1F+ strains |
| 27. | 1994 | S Ghosh, A Mittal, NK Ganguly | Purification and characterization of distinct type of mannose-sensitive fimbriae from *Salmonella typhimurium* | Typhimurium | 11828 | Temp: 37°C  Time: 60 h;  Medium: separately on colonization  factor antigen agar for 60 h and  BHI agar for 18 h at 37°C in 1-1 Roux bottles  O_2_: ?  Shaking: ? | Guinea-pig erythrocytes | MS agglutination |
| 28. | 1996 | MG Sojka, M Dibb-Fuller, CJ Thorns | Characterisation of monoclonal antibodies specific to SEF 21 fimbriae of *Salmonella enteritidis* and their reactivity with other Salmonellae and Enterobacteria | Enteritidis,  Isolates from serogroup B, C, D, E G-K | - | Temp: 37°C?  Time: 2 to 6 days  Medium: heart infusion broth  O_2_: ?  Shaking: ? | Horse erythrocytes | MS agglutination of T1F+ strains |
| 29. | 1996 | AJ Baumler, RM Tsolis. F Heffron | Contribution of fimbrial operons to attachment to and invasion of epithelial cell lines by *Salmonella typhimurium* | Typhimurium | IR715- a  derivative of ATCC 14028, SR-11 derivative  AJB3 (T1F+);  AJB4 (Δ*fim*, T1F-) | Temp: ?  Time: ?  Medium:?  O_2_: Aerobic?  Shaking: static | *Saccharomyces cerevisiae* | MS agglutination of T1F+ strains |
| 30. | 1996 | S Ghosh, A Mittal, H Vohra, NK Ganguly | Interaction of a rat intestinal brush border membrane glycoprotein with type-1 fimbriae of *Salmonella typhimurium*. | Typhimurium | 11828 | Temp: 37°C  Time: 18h  Medium: BHI agar  O_2_: ?  Shaking:? | Guinea-pig erythrocytes | MS agglutination |
| 31. | 1997 | SWB Ewen, Pj Naughton, G Grant, M Sojka, E Allen-Vercoe, S Bardocz, CJ Thorns, A Pusztai | *Salmonella enterica* var Typhimurium and *Salmonella enterica* var Enteritidis express type 1 fimbriae in the rat in vivo | Typhimurium, Enteritidis | Tym- S986, Ent- 857 | Temp: ?  Time: 48h  Medium: nutrient broth O_2_: ?  Shaking: static | Horse erythrocytes | MS agglutination |
|  | 1998 | LS Hancox, KS Yeh, S Clegg | Construction and characterization of type 1 non-fimbriate and non-adhesive mutants of *Salmonella typhimurium* | Typhimurium | LB5010 (LT2 derivate) | Temp: 37°C  Time: 24h or 48-72h  Medium: L-media  O_2_: ?  Shaking: ? | HeLa, HEp-2 | T1F-dependent adhesion |
| 32. | 1998 | MG Sojka, MA Carter, CJ Thorns | Characterisation of epitopes of type 1 fimbriae of Salmonella using monoclonal antibodies specific for SEF21 fimbriae of *Salmonella enteritidis* | Various serotypes | - | Temp: 37°C  Time: 48 h;  Medium: heart infusion broth  O_2_: ?  Shaking: static | Horse erythrocytes | Confirmation of T1F expression and mAb binding site |
| 33. | 1998 | M Kukkonen, S Saarela, K Lahteenmaki, U Hynonen,  B Westerlund-Wikstrom, M Rhen, TK Korhonen | Identification of two laminin-binding fimbriae, the type 1 fimbria of *Salmonella enterica* serovar Typhimurium and the G fimbria of *Escherichia coli*, as plasminogen receptors | Typhimurium | SH401 | Temp: ?  Time: ?;  Medium: Luria broth  O_2_: ?  Shaking: ? | *Saccharomyces cerevisiae* | MS agglutination |
| 34. | 1999 | K Thankavel, AH Shah, MS Cohen, T Ikeda, RG Lorenz,  R Curtiss III, SN Abraham | Molecular basis for the enterocyte tropism exhibited by *Salmonella typhimurium* type 1 fimbriae. | Typhimurium | SR-11 derivates- X 4252 (T1F+), 4253 (T1F-) | Temp: 37°C  Time: until  bacteria were in mid-log phase growth  Medium: LB  O_2_: ?  Shaking: static | *Candida albicans,* guinea-pig erythrocytes | MS agglutination of T1F+ strain |
| 35. | 1999 | E Allen-Vercoe, MJ Woodward | The role of flagella, but not fimbriae, in the adherence of *Salmonella enterica* serotype Enteritidis to chick gut explant. | Enteritidis | S1400/94 | Temp: 37°C  Time: 48 h;  Medium: Brain heart  infusion broth  O_2_: ?  Shaking: static | Guinea-pig erythrocytes | MS agglutination of T1F+ strain |
|  |  |  |  |  |  |  | Chicken gut explants | No T1F-dependent adhesion |
| 36. | 2001 | PJ Naughton, G Grant, S Bardocz, E Allen Vercoe, MJ Woodward, A Pusztai | Expression of type 1 fimbriae (SEF 21) of *Salmonella enterica* serotype Enteritidis in the early colonisation of the rat intestine. | Enteritidis | LA5 (T1F+); LA5 EAV3 (T1F-) | Temp: ?  Time: 48 h  Medium: nutrient broth  O_2_: ?  Shaking: static | Horse erythrocytes | MS agglutination of T1F+ strain |
| 37. | 2002 | JD Boddicker, NA Ledeboer, J Jagnow, BD Jones, S Clegg | Differential binding to and biofilm formation on, HEp-2 cells by *Salmonella enterica* serovar Typhimurium is dependent upon allelic variation in the *fimH* gene of the fim gene cluster. | Typhimurium | LB5010 (LT2 derivate), SL1344 (T1F+); LBH4 (*fimH* mutant of LB5010), SL1344H3 (*fimH* mutant of SL1344) (T1F-) | Temp: 37°C  Time: 48h  Medium: LB  O_2_: ?  Shaking: static | Guinea-pig erythrocytes | MS agglutination of T1F+ strain |
|  |  |  |  |  |  |  | HEp-2 | LB5010- T1F-dependent adhesion,  SL1344- no/very low T1F-dependent adhesion |
| 38. | 2006 | NA Ledeboer, JG Frye, M McClelland, BD Jones | *Salmonella enterica* serovar Typhimurium requires the Lpf, Pef, and Tafi fimbriae for biofilm formation on HEp-2 tissue culture cells and chicken intestinal epithelium. | Typhimurium | BJ2710- SL1344 derivative containing the LB5010 *fimH* gene (T1F+);  BJ2508- BJ2710 *fimH::kan* (T1F-) | Temp: 37°C  Time: 48 h;  Medium: 10 ml of LB broth  O_2_: ?  Shaking: static | Guinea-pig erythrocytes | MS agglutination of T1F+ strain |
| 39. | 2007 | A Guo, MA Lasaro, JC Sirard, JP Kraehenbuhl, DM Schifferli | Adhesin-dependent binding and uptake of *Salmonella enterica* serovar Typhimurium by dendritic cells | Typhimurium | AJB3- x4252 (NalR), derived from SR-11 (T1F+); AJB4- x4253 (NalR), derived from SR-11 Δ*fim* (T1F-) | Temp: 37°C  Time: 48 h;  Medium:  O_2_: ?  Shaking: static | *Saccharomyces cerevisiae* | MS agglutination of T1F+ strain |
|  |  |  |  |  |  |  | Murine bone-marrow-derived dendritic cells | MS adhesion and internalization of T1F+ strain |
| 40. | 2009 | A Guo, S Cao, L Tu, P Chen, C Zhang, A Jia, W Yang, Z Liu, H Chen, DM Schifferli | FimH alleles direct preferential binding of Salmonella to distinct mammalian cells or to avian cells | Typhimurium,  Gallinarum | Isogenic model with expression of Typhimurium FimH variants from: AJB3, SL1344, AZb62 and various derivates | Temp: 37°C  Time: 48 h;  Medium:  O_2_: ?  Shaking: static | Murine bone-marrow-derived dendritic cells | MS adhesion and FimH variant dependent adhesion |
|  |  |  |  |  |  |  | HEp-2 | FimH variant dependent adhesion |
|  |  |  |  |  | AJB3, AZb57,  Isogenic model with expression of Typhimurium and Gallinarum FimH variants | Temp: 37°C  Time: 48 h;  Medium:  O_2_: ?  Shaking: static | Chicken leukocytes | FimH variant dependent adhesion (Gallinarum adheres better),  Typhimurium- MS adhesion  Gallinarum- MR adhesion |
| 41. | 2009 | L Borowsky, G Corcao, M Cardoso | Mannanoligosaccharide agglutination by *Salmonella enterica* strains isolated from carrier pigs. | 26 serovars | 108 isolates | Temp: 37°C  Time: overnight  Medium: Clumping Factor Agar  O_2_: ?  Shaking: static | Guinea-pig erythrocytes | MS agglutination of 31 isolates |
| 42. | 2011 | BE Dwyer, KL Newton, D Kisiela, EV Sokurenko, S Clegg | Single nucleotide polypmorphisms of fimH associated with adherence and biofilm formation by serovars of Salmonella enterica | 14 serovars | 45 strains | Temp: 37°C  Time: 48 h periods  Medium: 10 ml LB broth  O_2_: ?  Shaking: static | Guinea-pig erythrocytes | MS agglutination of 23 isolates |
|  |  |  |  |  |  |  | HEp-2 | FimH variant dependent adhesion |
| 43. | 2012 | M Kuźmińska-Bajor, M Kuczkowski, K Grzymajło, Ł Wojciech, M Sabat, D Kisiela, A Wieliczko, M Ugorski | Decreased colonization of chicks by *Salmonella enterica* serovar Gallinarum expressing mannose-sensitive FimH adhesin from *Salmonella enterica* serovar Enteritidis | Gallinarum, Enteritidis | Gallinarum- isolate no. 589/02 (1); Δ*fimH* mutant (2); Gallinarum with Enteritidis FimH variant (3) | Temp: 37°C  Time: 5 SP  Medium: LB broth  O_2_: ?  Shaking: static | Chicken leukocytes | 1- MR high adhesion  2- MR low adhesion  3- MS high adhesion |
| 44. | 2012 | SA Zeiner, BE Dwyer, S Clegg | FimA, FimF, and FimH are necessary for assembly of type 1 fimbriae on *Salmonella enterica* serovar Typhimurium | Typhimurium | SL1344 (T1F+); various mutant derivates of SL1344 strain () | Temp: 37°C  Time: serially grown  Medium: 10-ml broth cultures  O_2_: ?  Shaking: static | Guinea-pig erythrocytes | MS agglutination of T1F+ strain |
| 45. | 2016 | KC Wang, CH Huang, SM Ding, CK Chen, HW Fang, MT Huang, SB Fang | Role of *yqiC* in the Pathogenicity of Salmonella and Innate Immune Responses of Human Intestinal Epithelium | Typhimurium | SL1344 WT (T1F-); SL1344 Δ*yqiC* (T1F+) | Temp: 28°C and 37°C  Time: streaked  on LB agar plates then incubated for 18 h  Medium: LB  O_2_: ?  Shaking: static | *Saccharomyces cerevisiae* | MS agglutination of T1F+ strain |
| 46. | 2016 | CA Lee, KS Yeh | The Non-Fimbriate Phenotype Is Predominant among *Salmonella enterica* Serovar Choleraesuis from Swine and Those Non-Fimbriate Strains Possess Distinct Amino Acid Variations in FimH. | Choleraesuis | 120 isolates | Temp: 28°C and 37°C  Time: 48h  Medium: 10-mL of LB broth  O_2_: ?  Shaking: static | *Saccharomyces cerevisiae* | MS agglutination of 4 isolates |

Supplementary Table 1. Summary of adhesion and/or invasion assays performed with cells and organ explants to study role of T1F in *Salmonella* virulence.

Abbreviations used in the table: Temp.- Temperature; O_2_- presence of oxygen during growth; MR- mannose resistant; MS- mannose sensitive; T1F- type 1 fimbriae; “T1F+”- type 1 fimbriae expressing; “T1F-”- type 1 fimbriae non-expressing;
