# Supplementary material for: Everything You Always Wanted to Know About Salmonella Type 1 Fimbriae, but Were Afraid to Ask
Source: Front Microbiol. 2019 May 14;10:1017. doi: 10.3389/fmicb.2019.01017 (PMC6527747; doi:10.3389/fmicb.2019.01017)
Supplement: Supplementary file 2 [file Table_2.DOCX]

Supplementary Table 2. FimH sequence variation in *Salmonella*. Amino-acids identical to the amino-acids of first sequence are marked by dots. Dashes indicate sites with no amino-acid aligned. The site numbers on the top of the table correspond to position of of variable site in FimH multiple alignment.

| Site  Serovar | 2 | 13 | 15 | 32 | 50 | 57 | 58 | 61 | 63 | 71 | 74 | 78 | 89 | 101 | 118 | 126 | 127 | 128 | 131 | 137 | 158 | 159 | 162 | 166 | 177 | 182 | 217 | 222 | 224 | 230 | 235 | 245 | 254 | 267 | 278 | 279 | 280 | 285 | 289 | 318 | 321 |
| --- | --- | --- | --- | --- | --- | --- | --- | --- | --- | --- | --- | --- | --- | --- | --- | --- | --- | --- | --- | --- | --- | --- | --- | --- | --- | --- | --- | --- | --- | --- | --- | --- | --- | --- | --- | --- | --- | --- | --- | --- | --- |
| Typhimurium | K | A | F | A | P | P | E | G | V | A | T | T | Q | N | F | L | M | G | Y | K | N | M | I | T | G | T | S | A | A | G | T | V | R | S | N | S | N | T | - | I | A |
| Typhimurium | . | . | . | . | . | . | . | A | . | . | . | . | . | . | S | . | . | . | . | . | . | . | . | . | . | . | . | . | . | . | . | . | . | . | . | . | . | . | - | . | . |
| Typhimurium | . | . | . | . | . | . | . | . | . | . | . | . | . | . | . | . | . | . | . | . | . | . | . | R | . | . | . | . | . | . | . | . | . | . | . | . | . | . | - | . | . |
| Typhimurium | . | . | . | . | . | . | . | . | . | . | . | . | . | . | . | . | . | . | . | . | . | . | . | . | . | . | . | . | . | . | . | A | . | . | . | . | . | . | - | . | . |
| Typhimurium | . | . | . | . | . | . | . | . | . | . | . | . | . | . | . | . | . | . | . | . | Y | . | . | . | . | . | . | . | . | . | . | . | . | . | . | . | . | . | - | . | . |
| Typhimurium | . | . | . | . | . | . | . | A | . | . | . | . | . | . | S | . | . | . | . | . | . | . | . | . | . | . | . | . | . | . | . | . | . | . | . | . | . | . | - | . | . |
| Choleraesuis | . | . | . | . | S | . | . | . | . | . | . | . | . | . | . | . | . | . | S | . | . | . | . | . | . | . | . | . | . | . | . | . | . | . | . | G | . | . | - | N | . |
| Choleraesuis | . | . | . | . | . | L | . | . | G | . | . | . | R | . | . | R | . | . | S | . | . | . | . | . | . | . | . | . | . | . | . | . | . | . | . | . | . | . | - | N | . |
| Choleraesuis | . | . | . | . | . | L | . | . | . | . | . | . | R | . | . | R | . | . | S | . | . | . | . | . | . | . | . | . | . | . | . | . | . | . | . | . | . | . | - | N | . |
| Dublin | . | . | . | . | . | . | . | . | . | . | . | . | . | S | . | R | . | . | S | M | . | . | . | . | . | . | . | . | . | . | . | . | . | . | . | . | . | I | - | N | . |
| Enteritidis | . | . | . | . | . | . | . | . | . | . | . | . | . | . | . | R | . | . | S | M | . | . | . | . | . | . | . | . | . | . | . | . | . | . | . | . | . | . | - | N | . |
| Gallinarum biovar Pullorum | . | T | . | . | . | . | . | . | . | . | . | I | . | . | . | R | . | . | S | M | . | . | . | . | . | . | . | . | . | . | . | . | . | . | . | . | . | . | - | N | . |
| Gallinarum biovar Pullorum | . | T | . | . | . | . | . | . | . | . | . | I | . | . | . | R | . | . | S | M | . | . | . | . | . | . | . | V | . | . | . | . | . | . | . | . | . | . | - | N | . |
| Gallinarum biovar Gallinarum | . | . | . | . | . | . | . | . | . | . | . | I | . | . | . | R | . | . | S | M | . | . | . | . | . | S | . | V | . | . | . | . | . | . | . | . | . | . | - | N | . |
| Javianna | . | . | . | V | . | . | . | . | . | . | . | . | R | . | . | R | . | . | S | M | . | . | . | . | . | . | . | . | . | . | . | . | . | . | . | . | . | . | - | N | . |
| Pomona | . | . | . | V | . | . | . | . | . | . | . | . | R | . | . | R | . | . | S | M | . | . | . | . | . | . | . | . | . | . | . | . | . | . | . | . | . | . | - | N | . |
| Limete | . | . | . | V | . | . | . | . | . | . | . | . | R | . | . | R | . | . | S | M | . | . | . | . | . | . | . | . | . | . | . | . | . | . | . | . | . | . | - | N | . |
| Poona | . | . | . | V | . | . | . | . | . | . | . | . | R | . | . | R | . | . | S | M | . | . | . | . | . | . | . | . | . | . | . | . | . | . | . | . | . | . | - | N | . |
| Anatum | . | . | . | . | . | . | . | . | . | E | M | . | R | . | . | R | . | . | S | M | . | . | . | . | . | . | . | . | . | . | . | . | . | . | . | . | . | . | - | . | . |
| Mississippi | . | . | . | . | . | . | . | . | . | . | . | . | . | . | . | . | . | . | S | . | . | . | . | . | . | . | . | . | . | . | . | . | . | . | . | . | . | . | - | N | V |
| Agona | . | . | . | V | . | . | . | . | . | . | . | . | . | . | . | R | . | . | S | M | . | . | . | . | . | . | . | . | . | . | . | . | . | . | . | . | . | . | - | N | . |
| Paratyphi B | . | . | . | . | . | . | . | . | . | . | . | I | . | . | . | R | . | . | S | M | . | . | . | . | . | . | . | . | . | . | . | . | . | . | . | . | . | . | N | . | . |
| Paratyphi B | . | . | . | . | . | . | . | . | . | . | . | . | . | . | . | R | I | . | S | M | . | . | . | . | . | . | . | . | . | . | . | . | . | . | . | . | . | . | N | . | . |
| Paratyphi B | . | . | . | . | . | . | . | . | . | . | . | . | . | . | . | R | . | D | S | M | . | . | . | . | . | . | . | . | . | . | . | . | . | . | . | . | . | . | N | . | . |
| Paratyphi B var java | . | . | . | . | . | . | . | . | . | . | . | . | . | . | . | R | . | . | S | M | . | . | . | . | . | . | . | . | . | . | . | . | . | . | . | . | . | . | - | . | . |
| Paratyphi C | . | . | . | . | . | . | . | . | . | . | . | . | R | . | . | R | . | . | S | . | . | . | . | . | . | . | . | . | . | . | . | . | W | . | . | . | . | . | - | N | . |
| Typhisuis | E | . | . | . | . | . | . | . | . | . | . | . | R | . | . | R | . | . | S | . | . | . | . | . | . | . | . | . | . | . | . | . | . | . | . | . | . | . | - | N | . |
| Typhisuis | . | . | . | . | . | . | . | . | . | . | . | . | R | . | . | R | . | . | S | . | . | . | . | . | . | . | . | . | . | . | . | . | . | . | . | . | . | . | - | N | . |
| Typhi | . | . | . | . | . | L | K | D | C | . | . | . | . | . | . | R | . | . | S | . | . | I | . | . | . | . | P | . | . | . | A | . | . | . | . | . | . | . | - | N | . |
| Sendai | . | . | . | . | . | . | . | . | . | . | . | . | . | . | . | . | . | . | . | . | . | . | . | . | . | . | . | . | . | . | A | . | . | . | . | . | . | . | - | N | . |
| Paratyphi A | . | . | . | . | . | . | . | . | . | . | . | . | . | . | . | . | . | . | . | . | . | . | . | . | . | . | . | . | . | . | A | . | . | . | . | . | . | . | - | N | . |
| Muenchen | . | S | . | . | . | . | . | . | . | . | . | . | R | . | . | . | . | . | . | . | . | . | . | . | S | . | . | . | . | . | . | . | . | . | . | . | S | . | - | N | . |
| Muenchen | . | . | . | . | . | . | . | . | . | . | . | . | R | . | . | . | . | . | . | . | . | . | . | . | S | . | . | . | . | . | . | . | . | . | . | . | S | . | - | N | . |
| Montevideo | . | . | . | . | . | . | . | . | . | . | . | . | R | . | . | R | . | . | S | M | . | . | . | . | . | . | . | . | . | . | . | . | . | . | . | . | S | . | - | N | . |
| Montevideo | . | S | . | . | . | . | . | . | . | . | . | . | R | . | . | . | . | . | . | . | . | . | . | . | S | . | . | . | . | . | . | . | . | . | . | . | S | . | - | N | . |
| Indiana | . | . | . | . | . | . | . | . | . | . | . | . | R | . | . | R | . | . | S | . | . | . | . | . | . | . | . | . | . |  | . | . | . | . | . | . | . | . | - | N | . |
| Wien | . | . | . | . | . | . | . | . | . | E | M | . | R | . | . | R | . | . | S | M | . | . | . | . | . | . | . | . | . | . | . | . | . | . | . | . | . | . | - | N | . |
| Panama | . | . | . | . | . | . | . | . | . | . | . | . | R | . | . | R | . | . | S | M | . | . | . | . | . | . | . | . | . | . | . | . | . | . | . | . | . | . | - | N | . |
| Sandiego | . | . | . | . | . | . | . | . | . | . | . | . | . | . | . | . | . | . | . | . | . | . | . | . | . | . | . | . | . | . | . | . | . | . | . | . | . | . | - | N | V |
| Hadar | . | . | . | V | . | . | . | . | . | . | . | . | . | . | . | R | . | . | S | M | . | . | . | . | . | . | . | . | . | . | . | . | . | . | . | . | . | . | - | N | . |
| Newport | . | . | . | . | . | . | . | . | . | . | . | . | R | . | . | R | . | . | S | M | . | . | . | . | . | . | . | . | . | . | . | . | . | . | . | . | . | . | - | N | V |
| Newport | . | . | L | V | . | . | . | . | . | . | . | . | . | . | . | R | . | . | S | M | . | . | . | . | . | . | . | . | . | S | . | . | . | . | . | . | . | . | - | N | V |
| Abortusequi | . | . | . | V | . | . | . | . | . | . | . | . | . | . | . | R | . | . | S | M | . | . | L | . | . | . | . | . | . | . | . | . | . | . | . | . | . | . | - | N | V |
| Newport | . | . | L | V | . | . | . | . | . | . | . | . | . | . | . | R | . | . | S | M | . | . | . | . | . | . | . | . | . | . | . | . | . | . | . | . | . | . | - | N | V |
| Abortusovis | . | . | . | . | . | . | . | . | . | . | . | . | . | . | . | R | . | . | S | . | . | . | . | . | . | . | . | . | . | . | A | . | . | . | . | . | . | . | - | N | . |
| Abortusovis | . | . | . | . | . | . | . | . | . | . | . | . | . | . | . | C | . | . | S | . | . | . | . | . | . | . | . | . | . | . | A | . | . | . | . | . | . | . | - | N | . |
