# Supplementary material for: Everything You Always Wanted to Know About Salmonella Type 1 Fimbriae, but Were Afraid to Ask
Source: Front Microbiol. 2019 May 14;10:1017. doi: 10.3389/fmicb.2019.01017 (PMC6527747; doi:10.3389/fmicb.2019.01017)
Supplement: Supplementary file 3 [file Presentation_1.pdf]

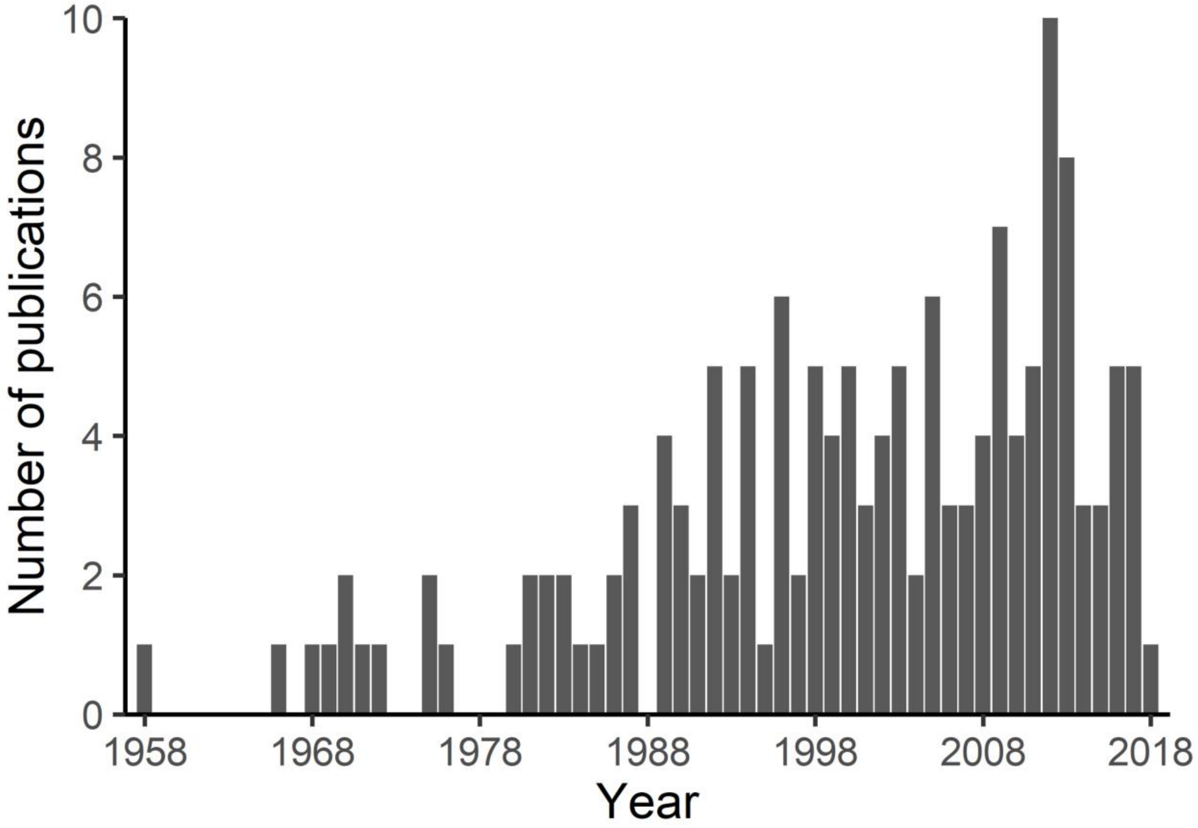

Supplementary Fig. 1. **Prevalence of publications about *Salmonella* T1F.** All publications concerning investigations about T1F in *Salmonella* were counted. The graph shows the number of publications per year. XXX publications found from 1958 to 2018. The X-axis shows the year of publication and the Y-axis shows the number of publications.
